# Hair glucocorticoids are associated with childhood adversity, depressive symptoms and reduced global and lobar grey matter in Generation Scotland

**DOI:** 10.1038/s41398-021-01644-9

**Published:** 2021-10-12

**Authors:** Claire Green, Aleks Stolicyn, Mathew A. Harris, Xueyi Shen, Liana Romaniuk, Miruna C. Barbu, Emma L. Hawkins, Joanna M. Wardlaw, J. Douglas Steele, Gordon D. Waiter, Anca-Larisa Sandu, Archie Campbell, David J. Porteous, Jonathan R. Seckl, Stephen M. Lawrie, Rebecca M. Reynolds, Jonathan Cavanagh, Andrew M. McIntosh, Heather C. Whalley

**Affiliations:** 1 Division of Psychiatry, University of Edinburgh, Edinburgh, UK; 2 UK Dementia Research Institute, Edinburgh Medical School, University of Edinburgh, Edinburgh, UK; 3 Centre for Clinical Brain Sciences, University of Edinburgh, Edinburgh, UK; 4 Division of Imaging Science and Technology, School of Medicine, University of Dundee, Dundee, UK; 5 Aberdeen Biomedical Imaging Centre, Institute of Medical Sciences, University of Aberdeen, Aberdeen, UK; 6 Centre for Genomic and Experimental Medicine, Institute of Genetics and Cancer, University of Edinburgh, Edinburgh, UK; 7 Centre for Cardiovascular Science, Queen’s Medical Research Institute, University of Edinburgh, Edinburgh, UK; 8 Institute of Infection, Immunity & Inflammation, College of Medical and Veterinary Life Sciences, University of Glasgow, Glasgow, UK

**Keywords:** Depression, Neuroscience

## Abstract

Hypothalamic–pituitary–adrenal (HPA) axis dysregulation has been commonly reported in major depressive disorder (MDD), but with considerable heterogeneity of results; potentially due to the predominant use of acute measures of an inherently variable/phasic system. Chronic longer-term measures of HPA-axis activity have yet to be systematically examined in MDD, particularly in relation to brain phenotypes, and in the context of early-life/contemporaneous stress. Here, we utilise a temporally stable measure of cumulative HPA-axis function (hair glucocorticoids) to investigate associations between cortisol, cortisone and total glucocorticoids with concurrent measures of (i) lifetime-MDD case/control status and current symptom severity, (ii) early/current-life stress and (iii) structural neuroimaging phenotypes, in *N* = 993 individuals from Generation Scotland (mean age = 59.1 yrs). Increased levels of hair cortisol were significantly associated with reduced global and lobar brain volumes with reductions in the frontal, temporal and cingulate regions (*β*_range_ = −0.057 to −0.104, all *P*_FDR_ < 0.05). Increased levels of hair cortisone were significantly associated with MDD (lifetime-MDD status, current symptoms, and severity; *β*_range_ = 0.071 to 0.115, all *P*_FDR_ = < 0.05), with early-life adversity (*β* = 0.083, *P* = 0.017), and with reduced global and regional brain volumes (global: *β* = −0.059, *P* = 0.043; nucleus accumbens: *β* = −0.075, *P*_FDR_ = 0.044). Associations with total glucocorticoids followed a similar pattern to the cortisol findings. In this large community-based sample, elevated glucocorticoids were significantly associated with MDD, with early, but not later-life stress, and with reduced global and regional brain phenotypes. These findings provide important foundations for future mechanistic studies to formally explore causal relationships between early adversity, chronic rather than acute measures of glucocorticoids, and neurobiological associations relevant to the aetiology of MDD.

## Introduction

Major depressive disorder (MDD) is the leading cause of disability worldwide [1] and affects ~6% of the adult population globally per year [2]. Exposures to psychosocial stress and stressful circumstances are consistently implicated in the aetiology of MDD and are associated with onset, severity, remission, and antidepressant response [3, 4]. Early-life stress in particular is one of the largest environmental risk factors for depression [5], and the association between childhood adversity and subsequent psychopathology has been linked to dysregulation of the hypothalamic–pituitary–adrenal (HPA) axis [6–10]. However, precise mechanisms in MDD are unclear since previous findings are inconsistent with substantial variability in effect sizes across studies [11–16]. This is potentially due to differing approaches to the measurement of the highly phasic/variable HPA system, which is predominantly measured using cross-sectional measures of glucocorticoids in blood, saliva or spot urine samples. These acute measures demonstrate strong diurnal effects and both inter- and intra-individual fluctuations in response to environmental factors and are therefore temporally unstable [17]. Further, the structural neural correlates of prolonged glucocorticoid exposure in the context of MDD and in relation to early-life stress remains unclear.

Glucocorticoids are the liposoluble downstream effectors of the HPA-axis and can readily cross the blood–brain barrier. Murine models indicate that long-term excess glucocorticoid exposure suppresses neurogenesis, decreases dendritic branching and inhibits synaptogenesis, most notably in the hippocampus [18–20]. Previous human studies of glucocorticoid effects on the brain have typically relied on acute cross-sectional measures from saliva, blood, or urine. Although findings should be interpreted cautiously due to the short timeframe and variability of these measures, these studies to some extent indicate that elevated levels of cortisol are associated with general global brain atrophy [21, 22]. One study of urinary markers also found that elevated glucocorticoid levels at baseline predicted subsequent brain atrophy and cognitive decline over the following 6 years [23]. Further, individuals with Cushing’s syndrome, characterised by long-term hypercortisolaemia, have been consistently shown to have structural brain abnormalities, as well as a high prevalence of cognitive deficits and low mood/depression [24–26].

Previous studies of glucocorticoid associations with brain structure in the context of MDD are however inconsistent, not only because of the prevailing use of temporally unstable serum/saliva measures, but they have also typically focused on one imaging modality (structural T1 weighted imaging), single regions of interest (e.g. the hippocampus), and with relatively small sample sizes (*N* < 50) [27–30]. Since there are no previous studies of long-term glucocorticoid exposure with both global/regional brain morphology and white matter microstructure phenotypes, there is a clear need for research that examines neuroarchitecture more broadly, and with measures that capture chronic glucocorticoid associations.

Hair glucocorticoid measures have been shown to provide a more temporally stable measure of exposure over several weeks compared to phasic blood/saliva measures [31–34]. However, there have been no prior studies of the association between hair glucocorticoids, structural neuroimaging phenotypes and depression. Furthermore, cortisone, the inert metabolite of cortisol, which is more prevalent in hair [35], has not been investigated in relation to brain imaging phenotypes, and may be a biologically relevant marker in the investigation of longer-term HPA-axis activity. Cortisone is activated to cortisol by 11β-hydroxysteroid dehydrogenase (11β-HSD) type 1 in target organs including the brain, liver, adipose tissue and vasculature. In contrast, 11β-HSD type 2 inactivates cortisol to cortisone, predominantly in the kidney, colon and salivary/sweat glands. Measuring both steroids in hair gives a more comprehensive measure of total glucocorticoid exposure over time.

In the current study, we report a large-scale investigation of hair glucocorticoid associations with brain structure and MDD in a large community-based sample (*N* = 993) from a cohort of deeply phenotyped individuals (Generation Scotland) in mid-late life. Previous work in this cohort has found that depression case/control status is associated with reduced total grey matter volumes, however, the degree to which HPA-axis activity plays a role in this association is currently unclear [36]. We sought to characterise structural neural correlates of HPA-axis dysregulation and associations with early-life adversity, current-life stress and depressive symptomatology. We utilised hair glucocorticoid measures of active cortisol, its inert metabolite cortisone and their total as markers of cumulative HPA-axis activity over the preceding weeks and months. In terms of neuroimaging, we investigated 190 structural neuroimaging-derived phenotypes using an a priori unbiased approach to characterise brain structure associations, including T1 and diffusion tensor imaging (DTI) measures. We investigated hair glucocorticoid associations with (i) MDD case/control status and depressive symptoms, (ii) childhood adversity and current-life stress and (iii) structural neuroimaging-derived phenotypes from the two imaging modalities.

Given the evidence from salivary/serum glucocorticoid measures described above, we hypothesised that increased hair cortisol, cortisone and their total would be related to MDD status and to increased measures of depressive symptoms, along with childhood adversity. We further hypothesised that increased glucocorticoids in hair would be associated with decreased global cortical volumes, regionally decreased hippocampal volumes and decreased global white matter microstructure integrity, in line with previous research.

## Materials and methods

### Participants

Participants in this study were recruited through Generation Scotland and included ~1000 individuals who were re-contacted in 2015–2019 for further assessment of mental health and brain imaging. Full details of the recruitment and demographics of this cohort are published elsewhere [36–38]. Demographics of the current sample are included in Table 1. In the current study, *N* = 993 individuals were included in symptom analyses, *N* = 894 individuals had T1 imaging data and *N* = 864 also had DTI data.

**Table 1:** Participant demographics.

Variable	Unit	Cases (*N* = 317)^c^	Controls (*N* = 676)^c^	*P*-value
Age^a^	Years (mean, SD)	57.4 (10.1)	60.8 (9.8)	<0.01
Sex^b^	Males (*N*)	72	265	<0.01
	Females (*N*)	245	411	
Study site^b^	Aberdeen	122	361	<0.01
	Dundee	195	315	
Hair processing batch^b^	Batch 1	228	506	0.367
	Batch 2	89	170	
Total QIDS score^a^	Mean (SD)	7.04 (4.9)	3.6 (2.3)	<0.01

^a^Wilcoxon *t*-test.

^b^Chi-squared test.

^c^Calculated by SCID diagnosis.

Ethical approval was formally obtained from the NHS Tayside committee on research (reference 14/SS/0039), and all participants provided written informed consent [37].

### Depression status and symptoms

We measured both the lifetime incidence of MDD (case/control status) and current depression symptoms and symptom severity. MDD case/control status was ascertained using the research version of the Structured Clinical Interview for DSM disorders (SCID) [39] and diagnostic criteria were based on the ‘Diagnostic and Statistical Manual of Mental Disorders’ (DSM-IV-TR). Using this definition, the sample had *N* = 317 MDD cases and *N* = 676 controls.

To assess depression symptoms, the ‘Quick Inventory of Depressive Symptomatology’ (QIDS) [40] was employed to assess both total current symptoms and symptom severity.

### Measures of early and current-life stress

Early-life stress was measured in terms of childhood trauma which was assessed using the ‘Childhood Trauma Questionnaire’ (CTQ), a retrospective 28-item questionnaire that assesses three areas of abuse (emotional, physical, and sexual) and two areas of neglect (emotional and physical) [41]. A total CTQ summary score was calculated as well as total scores for each subscale, with higher scores representing higher reported trauma [37].

Recent life stress was measured using a brief life events questionnaire: the ‘List of Threatening Experiences’ (LTE) [42], a self-report measure consisting of 12 questions regarding common and life-threatening events in the 6 months preceding the assessment. Where a participant has experienced one of these life events, a follow-up question required rating the threat from 3 (very bad), 2 (moderately bad) to 1 (not too bad) [43]. A total sum score of the LTE was calculated for analysis purposes.

### Hair glucocorticoid measurement

Hair samples were collected from the posterior vertex region of the head as close to the scalp as possible [37]. Cortisol (F) and cortisone (E) concentrations were measured by LC–MS/MS, at the Technische Universität, Dresden using an established method and following a standard wash and steroid extraction procedure [44]. Cortisol and cortisone levels were expressed as pg/mg. Total hair glucocorticoids (F + E) were also calculated for analytic purposes.

### MRI acquisition and analyses

Details of the structural neuroimaging-derived phenotypes included in the sample have been reported in full previously [36]. Briefly, participants had 3T MRI scans at one of two sites - Aberdeen or Dundee. Only the T1 and DTI data are used in this current study and 190 structural neuroimaging phenotypes were derived from the scans. Full details of the neuroimaging-derived phenotypes are provided in Supplementary Materials on pages 2 and 3. Briefly, global/lobar and regional metrics were derived for 34 cortical and 8 subcortical regions with FreeSurfer version 5.3 [45]. The DTI data were processed to extract fractional anisotropy (FA) and mean diffusivity (MD) measures for 24 tracts as well as global measures derived from principal component analysis (see Supplementary Information page 2 and 3 for further details).

### Statistical analyses

Hair cortisol, cortisone and their total were log-transformed and outliers ± 3 standard deviations from the mean were removed for statistical analyses. All analyses were conducted using R (version 3.2.3). For all global and lobar measures and CTQ/LTE/MDD measures, a generalised linear model was applied (function ‘glm’ in R package ‘stats’). For all bilateral imaging-derived phenotypes (T1 and DTI), both sides of the brain were included in mixed-effect linear models (function ‘lme’ in R package ‘nlme’) correcting for hemisphere as a within-subject measure [46, 47].

For case/control, QIDS and CTQ/LTE analyses, age, sex, assessment centre and glucocorticoid lab batch were included as covariates. For all phenotypes derived with FreeSurfer, age, sex, assessment centre, glucocorticoid lab batch, imaging edits, imaging batch and standardised intracranial volume (ICV) were included as covariates. For DTI data, age, sex, assessment centre and lab batch were used as covariates. False discovery rate (FDR) multiple comparison correction was applied per biomarker and per measure/modality to all depression measures, CTQ subscales, bilateral/regional structures, lobes and white matter tracts. Corrected *P*-values are referred to as *P*_FDR_ in this report and were obtained using the ‘p.adjust’ function in R, and all betas were standardised. FDR correction was not applied to global imaging metrics and to the summary measure of the CTQ and LTE, since these are representative singular measures of each of these phenotypes.

We investigated hair glucocorticoid associations with (i) MDD case/control status and QIDS scores, (ii) CTQ/LTE scores and (iii) structural neuroimaging-derived phenotypes.

### Relatedness analyses

As Generation Scotland is comprised of related individuals, we ran additional analyses excluding related individuals by randomly including one person per family for all of the brain imaging analyses. Randomisation was conducted in R using the ‘rnorm’ function to create a random seed variable for each participant and one individual per family with the highest random number was included in subsequent analyses, excluding all other family members. The unrelated dataset comprised *N* = 665 unrelated individuals with T1 data and *N* = 640 with DTI data.

## Results

### Demographics

Demographics and descriptive statistics of the key variables are presented in Table 1. Hair cortisol and cortisone were also positively correlated (*r* = 0.66, *P* < 0.001).

### Hair glucocorticoid associations with measures of depression

**Fig. 1:**
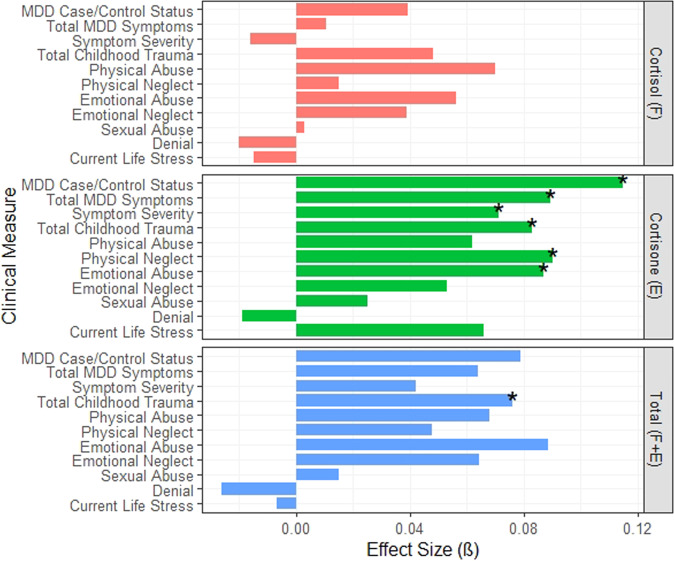
Standardised effect sizes (*ß*) of hair cortisol (F), cortisone (E) and total glucocorticoid associations (F + E) with depression and stress measures. MDD case/control Status is determined through SCID diagnosis. MDD symptom totals are total QIDS scores and the severity is also determined by QIDS scores. Trauma scores are determined from CTQ scores and current life stress is measured from LTE scores. Associations where *P* < 0.05 are marked with an asterisk.

We tested the associations between the three glucocorticoid measures and measures of depression (case/control status, total QIDS scores and QIDS severity; Fig. 1 and Supplementary Table 1). Although there were no FDR-significant associations with hair cortisol, increased hair cortisone concentrations were significantly associated with MDD case–control status (*β* = 0.115, *P*_FDR_ = 0.002), total QIDS scores (*β* = 0.089, *P*_FDR_ = 0.014), and QIDS depression severity (β = 0.071, *P*_FDR_ = 0.038). There were no FDR-significant associations between total glucocorticoid (F + E) concentrations and any measure of depression.

### Hair glucocorticoid associations with stress measures

We tested the associations between the three glucocorticoid measures with early-life adversity using total CTQ scores and the six subscales of the questionnaire (Supplementary Table 1). While we found no associations with hair cortisol, increased hair cortisone concentrations were significantly associated with total CTQ scores (*β* = 0.083, *P* = 0.017) and with two subscales of the CTQ: higher reported total emotional abuse (*β* = 0.087, *P*_FDR_ = 0.034) and total physical neglect (*β* = 0.090, *P*_FDR_ = 0.034). Total hair glucocorticoids (F + E) were also significantly associated with total CTQ scores (*β* = 0.076, *P* = 0.040) but not with any of the individual subscales. None of the hair glucocorticoid measures were significantly associated with recent life stress (as measured by total LTE scores).

### Hair glucocorticoid associations with global and lobar measures of brain structure

**Fig. 2:**
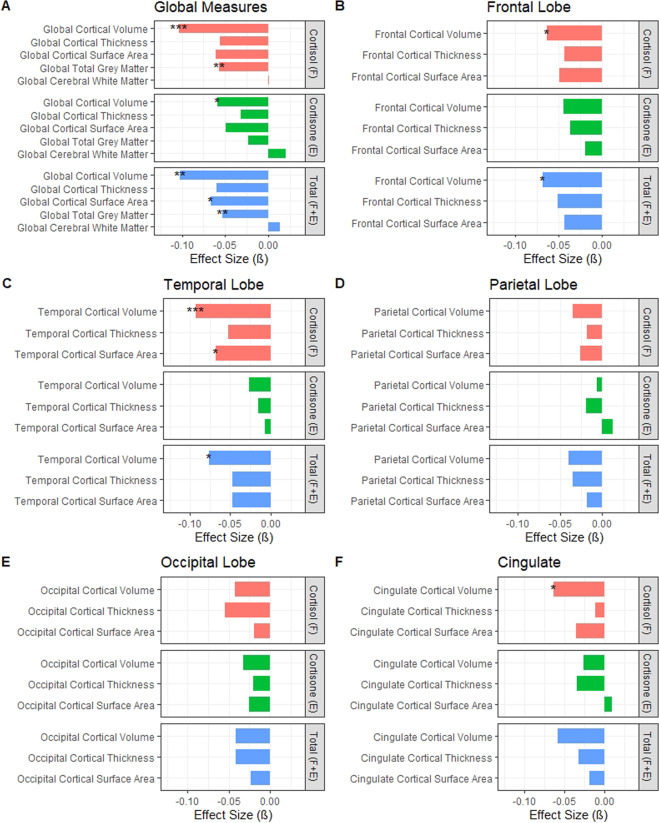
Standardised effect sizes (*ß*) of hair cortisol (F), cortisone (E) and total glucocorticoid (F + E) associations with global and lobar structural neuroimaging phenotypes. **A** Global neuroimaging measures, **B** frontal lobe neuroimaging measures, **C** temporal lobe neuroimaging measures, **D** parietal lobe neuroimaging measures, **E** occipital lobe neuroimaging measures, and **F** cingulate neuroimaging measures. *P < 0.05, **P < 0.01, ***P < 0.001.

Increased hair cortisol concentrations were significantly associated with reduced global grey matter volumes (*β* = −0.057, *P* = 0.003; Fig. 2) and reduced global cortical volumes (*β* = −0.104, *P* = 0.0007; Supplementary Table 2). This global loss was also reflected in regional reductions where higher hair cortisol was significantly associated with reduced cortical volumes in frontal (*β* = −0.064, *P*_FDR_ = 0.014), temporal (*β* = −0.093, *P*_FDR_ = 0.0005) and cingulate regions (*β* = −0.064, *P*_FDR_ = 0.040). Increased hair cortisol was also significantly associated with reduced surface area of the temporal lobe (*β* = −0.068, *P*_FDR_ = 0.042). Increased hair cortisone concentrations were significantly associated with reduced global cortical volume only (*β* = −0.059, *P* = 0.043). The total measure of hair glucocorticoids (F + E) was significantly associated with reduced total grey matter volumes (*β* = −0.053, *P* = 0.009), reduced global cortical volumes (*β* = −0.103, *P* = 0.001), and reduced global cortical surface area (*β* = −0.066, *P* = 0.0496). The total measure was also associated with decreased volume of the frontal (*β* = −0.068, *P*_FDR_ = 0.010) and temporal regions (*β* = −0.076, *P*_FDR_ = 0.010).

### Hair glucocorticoid associations with regional brain structures

**Fig. 3:**
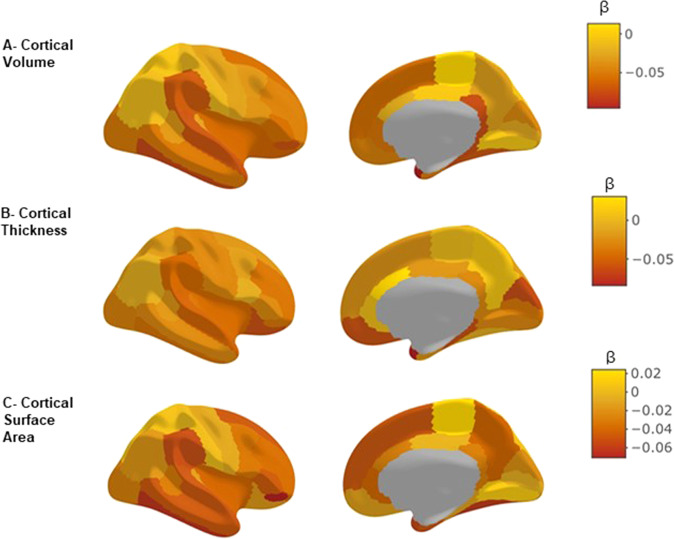
Brain map of the standardised effect sizes for hair cortisol associations with 34 regional cortical volumes, cortical thicknesses and cortical surface areas. **A** Cortical volume, **B** cortical thickness, and **C** cortical surface area. *ß* represents the standardised effect size. All associations are corrected for age, sex, study site, intracranial volume, imaging batch, imaging edits and hemisphere.

**Fig. 4:**
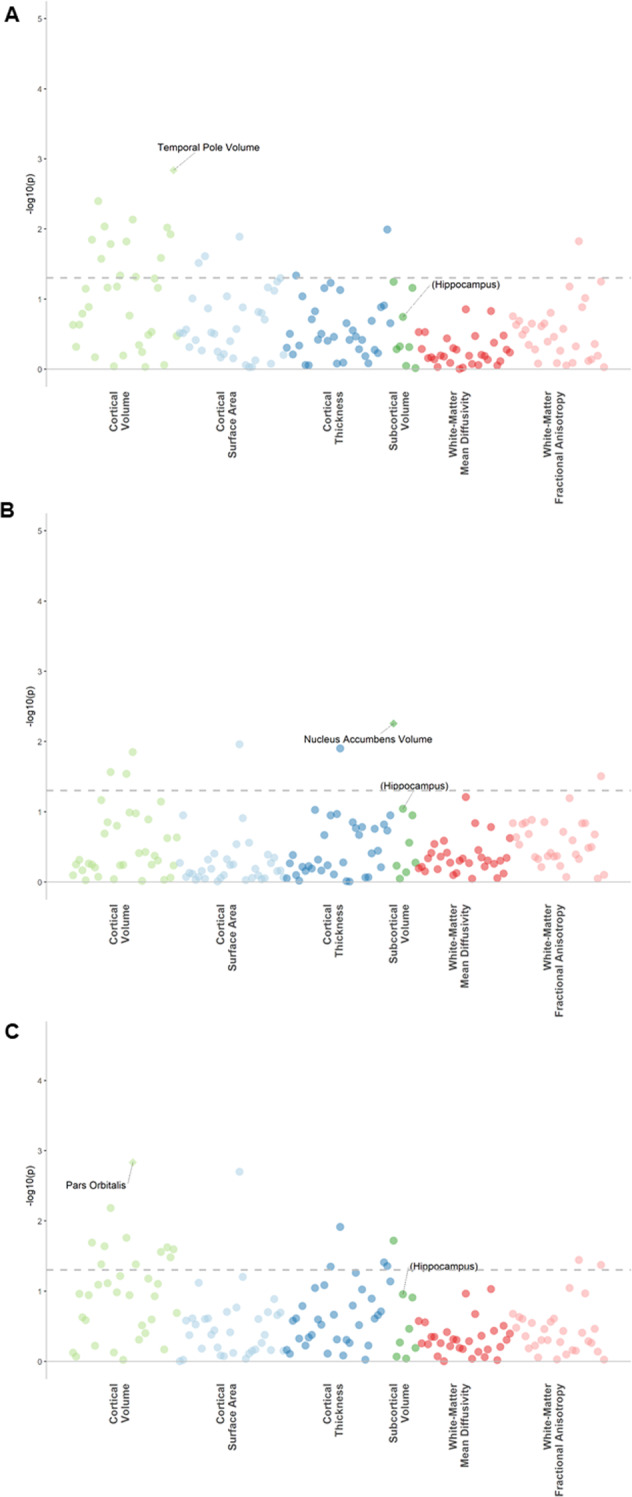
Significance plot for cortisol, cortisone and total glucocorticoid associations with regional structural imaging phenotypes. **A** Cortisol, **B** cortisone, and **C** total glucocorticoid. The x-axes represent structural imaging phenotypes, and the y-axes represent the −log10 of uncorrected p-values between the measure and the imaging phenotype corrected for covariates. Each dot represents one imaging phenotype, and the colours indicate their categories. The dashed lines indicate the *p*-value threshold of 0.05 and the diamonds represent phenotypes that survive FDR correction. The hippocampus (not significant) is marked in brackets for reference to previous studies.

We tested the associations of hair glucocorticoids with 34 cortical regions (volume, thickness and surface area measures) and 8 subcortical volumes and found that increased hair cortisol was significantly associated with reduced volume of the temporal pole (*β* = −0.096, *P*_FDR_ = 0.049; Supplementary Table 3) and also demonstrated consistently negative effect sizes for the 34 cortical measures although these did not reach corrected levels of significance (Fig. 3). We also found that increased hair cortisone concentrations were significantly associated with reduced volume of the nucleus accumbens (*β* = −0.075, *P*_FDR_ = 0.044; Fig. 4 and Supplementary Fig. 1, Supplementary Tables 4, 6). The measure of total hair glucocorticoids (F + E) was associated with reduced volume of the pars orbitalis (*β* = −0.089, *P*_FDR_ = 0.0497; Supplementary Fig. 2 and Supplementary Table 5). Both of these latter findings survived controlling for multiple comparisons.

### Hair glucocorticoid associations with white matter integrity

There were no significant associations after FDR correction between hair glucocorticoids and any measure of white matter integrity including global measures (Supplementary Tables 7–9).

### Relatedness analyses

All of the hair cortisol and total hair glucocorticoid (F + E) associations with global and lobar brain measures remained significant in the sample of unrelated participants (Supplementary Tables 10–17). For hair cortisone, the association with global cortical volume remained significant whereas the association with the nucleus accumbens did not, however, the effect size remained in the same direction and of a similar magnitude (*β*_unrelated_ = −0.06 versus *β*_related_ = −0.075). The total hair glucocorticoid association with the pars orbitalis also remained significant in the unrelated sample.

## Discussion

We report the findings from a comprehensive and large-scale, multimodal study with in-depth phenotypic data investigating hair glucocorticoid associations with brain morphology, measures of depressive psychopathology, and early/late life stress. Hair glucocorticoids were significantly associated with measures of depression, specific types of childhood trauma scores, but not with measures of current-life stress. In terms of brain morphology, hair glucocorticoids were associated with global reductions, along with regional volumetric loss in frontal, temporal and cingulate cortices, with some evidence for the involvement of reward-associated regions, but not with decreased hippocampal volumes, or with deficits in structural connectivity. This study provides important evidence in support of widely held hypotheses suggesting links between early adversity, disrupted HPA-axis functioning, altered brain morphology and depression, and lays essential foundations for future work to address causation and potential interventions.

Our findings expand on previous studies of acute salivary/serum measures of cortisol and MDD associations by utilising a hair measure of cumulative/integrated long-term glucocorticoid levels, and also additionally considering cortisone and total hair glucocorticoid associations [11–15]. Using these broader longer-term markers of HPA-axis activity, we found elevated levels of hair cortisone were significantly associated with three measures of depression: lifetime case/control status, total current depressive symptom scores and depression severity. The results, therefore, indicate that HPA-axis activity, as measured by hair cortisone, has a robust association with depression in terms of both current symptoms and lifetime incidence.

In addition, we also report that hair cortisone was associated with early-life stress, specifically in terms of childhood physical neglect and emotional abuse. Several previous studies have found that childhood adversity alters stress-reactivity in adulthood [48–53], with increased responses in individuals with depression [54]. One meta-analysis of 28 studies including *N* = 3397 individuals found that childhood adversity is also significantly related to hair cortisol concentrations and this association is moderated by the type and timing of the adversity experienced [55]. Animal work has also shown that early-life adversity/prenatal stress elicits changes in HPA-axis functioning that persists into adulthood (developmental programming), including HPA-axis hyperactivity and glucocorticoid/insulin resistance [56–58] and the frequent comorbidity between early-life adversity and MDD may be linked to HPA-axis dysregulation [59]. We build on this preclinical work in humans to show that chronically elevated glucocorticoids were specifically associated with early-life stress, but not contemporaneous stress, and were further specifically associated with childhood physical neglect and emotional abuse, as well as current and lifetime MDD. This finding further demonstrates the importance of the type and timing of adversity experienced with potential long-term consequences for HPA-axis functioning and wellbeing.

We further extend this work to also study associations with brain imaging phenotypes. To our knowledge, this is the first large-scale neuroimaging study of hair glucocorticoid associations with both T1 MRI and white matter microstructural integrity data in adults. We found higher concentrations of hair glucocorticoids were significantly associated with reductions in total grey matter and global cortical volumes. Increased hair cortisol concentrations were further associated with volumetric loss in the frontal/temporal lobes and cingulate regions. These areas are important for cognition and emotion regulation, and volumetric loss in these areas may underlie associations with MDD which is characterised by cognitive impairment and dysregulated emotional processing [60]. Preclinical work has shown that excess glucocorticoid exposure is associated with grey matter damage and that brain regions with higher numbers of glucocorticoid receptors are more vulnerable to these noxious effects including regions such as the cingulate described here [61–63]. Furthermore, animal models indicate that chronic glucocorticoid exposure is associated with changes in dendritic morphology, decreased neurogenesis/synaptogenesis and decreased plasticity, which may be indicative of the pathophysiological mechanisms underlying the grey matter loss seen in the current study [19, 64–67]. Taken together, these novel findings implicate chronically elevated glucocorticoids as having deleterious associations with brain structure which may potentially underlie associations with psychopathology, although formal tests of directionality are required.

In terms of regional findings, we also report associations between increased hair glucocorticoids and reduced volume of the nucleus accumbens and pars orbitalis (lateral/orbitofrontal cortex). Since these regions are central to reward processing, these findings suggest that elevated glucocorticoids are associated with structural alterations of reward neurocircuitry, which is also a key feature of MDD. This is consistent with previous studies which link chronic stress to altered reward processing by the attenuation of reward sensitivity, increasing vulnerability to anhedonia and psychopathology [68–71]. Animal work has also shown that glucocorticoid-receptor antagonists inhibit normal reward processing and that glucocorticoid neurotransmission plays a key role in reward-related behaviours [72, 73]. Future work should aim to replicate these associations in larger samples and further explore the importance of disrupted reward processing in the context of HPA-axis activity and early adversity in the aetiology of MDD.

Notably, we report no association between elevated glucocorticoids and hippocampal volumes, in contrast to previous findings [22, 27]. This may be due to our ‘non-ROI’ type approach, or to differing methods of measurement of HPA-axis activity. Hair measures may for example capture a distinct aspect of HPA-axis biology in relation to integrated long-term glucocorticoid secretion that may be specific to these imaging findings. We also note that we report differing patterns of associations dependent on whether we examine the active or the inactive metabolite. Hair cortisone was associated with clinical features and early-life adversity, while hair cortisol/ total hair glucocorticoids were primarily associated with neural features. Cortisol and cortisone are interconverted by two tissue-specific intracellular isozymes of 11β-HSD. Both glucocorticoids circulate at nanomolar levels in the blood. Whilst some have postulated that hair cortisol:cortisone reflects 11β-HSD in the hair follicle, there is little evidence of significant expression of either isozyme in this tissue. Thus, we consider it more probable that the specific hair glucocorticoid levels here reflect the kinetics of accumulation of cortisol and cortisone from the blood into the growing hair root.

In terms of limitations, our study was comprised of community-based, relatively well participants. Our findings may not, therefore, reflect associations of hair glucocorticoid measures in individuals with more severe forms of MDD but may be more widely generalisable to the population. The main findings of this sample were also conducted in a related sample, however, the additional analyses in the unrelated sample replicated the main findings of this paper, lending confidence to these findings. A further limitation is that we cannot exclude the possibility of biases in the retrospective self-report measures of childhood adversity in our sample and this should be considered when interpreting findings. Longitudinal research investigating childhood adversity, glucocorticoid trajectories and MDD would be able to disentangle these relationships further. In addition, given the association identified here between hair cortisone and MDD, future research in terms of causal directionality is clearly warranted utilising techniques such as Mendelian randomisation when suitable data of sufficient power is available, particularly as these findings may have important implications for the aetiology and treatment of depression. At the time of writing, the only large glucocorticoid genome-wide association studies available of sufficient power that we are aware of to independently generate the genetic instruments to test these causal relationships were based on plasma cortisol, rather than hair cortisone [74]. Future mechanistic work is also required to determine the underlying biology of the differential pattern of cortisol/ cortisone findings.

In conclusion, this study utilised a large sample with detailed behavioural phenotyping, multimodal imaging and chronic longer-term measures of glucocorticoids from hair samples. We found significant associations between elevated hair glucocorticoids and both current depression symptoms/severity and lifetime incidence. Prolonged glucocorticoid exposure was also associated with early-life adversity, specifically emotional abuse and physical neglect. Elevated hair glucocorticoids were robustly associated with global grey matter loss and volumetric lobar loss in frontal, temporal and cingulate regions. This regional loss was seen in areas of the brain that are important for cognition/emotion regulation, and in reward processing regions, and may potentially underlie associations with depression symptoms and severity. Although we cannot currently elucidate causal mechanisms, we have identified important relationships between longer-term measures of glucocorticoids, reduced grey matter volumes, and depression/early-life adversity. These findings are also consistent with preclinical work demonstrating long-term effects of early adversity on the HPA-axis, and deleterious effects of excess glucocorticoid exposure on the brain. This study also highlights the utility of hair measures of glucocorticoids as markers of longer-term HPA-axis activity. These findings, therefore, provide important foundations for future mechanistic studies to explore formal causal relationships between specific types of early adversity, prolonged glucocorticoid exposure, changes in brain morphology and subsequent psychopathology in order to develop novel and efficacious interventions.

## Supplementary information

Supplementary Materials

## Data Availability

The code of the statistical analyses is available from the corresponding author upon request.
