# Supplementary material for: Hair glucocorticoids are associated with childhood adversity, depressive symptoms and reduced global and lobar grey matter in Generation Scotland
Source: Transl Psychiatry. 2021 Oct 12;11:523. doi: 10.1038/s41398-021-01644-9 (PMC8511057; doi:10.1038/s41398-021-01644-9)
Supplement: Supplementary file 1 — Supplementary Materials [file 41398_2021_1644_MOESM1_ESM.docx]

**Hair glucocorticoids are associated with childhood adversity, depressive symptoms and reduced global and lobar grey matter in Generation Scotland**

**[Supplementary Materials]**

**CONTENTS:**

**MRI Parameters and Quality Control**

**Figures S1-S2:** Brain maps of standardized effect sizes for hair cortisone and total glucocorticoid (F+E) associations with 34 cortical regional measures of volume, thickness and surface area.

**Table S1:** Results of hair glucocorticoid associations with measures of (i) depression status and symptoms, (ii) current life stress (LTE sum score) and (iii) childhood trauma scores.

**Tables S2-S9:** Results of hair glucocorticoid associations with imaging phenotypes in the full related sample.

**Table S10-S17:** Results of brain imaging sensitivity analyses in an unrelated subsample (N=665 with T1 data and N=640 with DTI data).

**MRI Parameters**

Participants in Dundee were scanned using a Siemens 3T Prisma-FIT (Siemens Healthineers, Erlangen, Germany) with a 20-channel head and neck coil and a back-facing mirror (software version VE11, gradient with max amplitude 80 mT/m and maximum slew rate 200 T/m/s). In Aberdeen, participants were imaged on a 3T Philips Achieva TX series MRI system (Philips Healthcare, Best, Netherlands) with a 32-channel phased-array head coil with a back-facing mirror (software version 5.1.7); gradients with maximum amplitude 80 mT/m and maximum slew rate 100^1,2^. Both study centres followed the same protocol including structural sequences.

**FreeSurfer Quality Control**

All scans were visually assessed for parcellation errors and participants with any major errors in segmentation/cortical parcellation or those that had an infarct were excluded from analyses. Manual edits were made when the skull was included in parcellations to remove the skull from the parcellated tissue. Further edits were made if parts of the brain were not included in the parcellation such that we manually delineated the boundaries to include the missing tissue. Imaging site (Aberdeen/Dundee) and imaging batch were recorded to include in statistical analyses as the imaging data was processed in two batches. Additionally, scans were recorded as unedited (‘0’) versus edited (‘1’) to use as a covariate in statistical analyses as manual editing is a subjective process that may introduce bias.

**Diffusion Tensor Imaging Quality Control**

QC was performed following ENIGMA DTI protocols (http://enigma.ini.usc.edu/protocols/dti-protocols/). Briefly this included (1) correcting for eddy current-induced distortions and subject movement in the scanner; (2) skull stripping using BET at a threshold of 0.2; (3) using DTIFIT in order to compute diffusion tensor characteristics (i.e., principal eigenvectors or V1, V2, V3; eigenvalues or L1, L2, L3; fractional anisotropy (FA), mean diffusivity (MD); and (4) visually checking the quality of FA images at this stage in order to exclude distorted images. The 5 unilateral tracts included the corpus callosum, fornix and the body, genu and splenium of the corpus callosum^2^.

**Neuroimaging-derived Phenotypes**

T1 structural measures were processed using FreeSurfer version 5.3^3^ to quantify the volumes of 8 subcortical structures as well as the volumes, surface areas and thicknesses of 34 cortical regions per hemisphere according to the Desikan-Killany atlas^4^ (110 phenotypes). Global measures of grey matter volume, white matter volume, cortical surface area, thickness and volume were also derived (5 phenotypes). Lobar measures of volume, thickness and surface area of the frontal, parietal, temporal, occipital and cingulate regions were also calculated (15 phenotypes).

For DTI phenotypes, Tract Based Spatial Statistics (TBSS) was carried out according to the ‘Enhancing NeuroImaging Genetics through Meta-Analysis’ (ENIGMA) Consortium DTI protocol (http://enigma.ini.usc.edu/protocols/dti-protocols/). Region of interest (ROI) extraction analyses were then performed also using ENIGMA protocols to extract fractional anisotropy (FA) and mean diffusivity (MD) measures (<http://enigma.ini.usc.edu/protocols/dti-protocols/>). FA and MD measures were extracted for 5 unilateral tracts and 19 bilateral tracts according to the Johns-Hopkins University DTI-based white matter atlas^5^. Average FA and MD measures were also derived, which resulted in 50 phenotypes in total.

Global integrity was determined by applying principal component analysis (PCA) on the tracts to extract latent measures for FA and MD (2 phenotypes). Full details have been reported previously^2^. We then separately examined four subsets of white matter tracts for which scores of the first un-rotated principal component were also extracted for both FA/MD: (a) association fibres, (b) commissural fibres, (c) projection fibres and (d) thalamic radiations (8 phenotypes).

**Figure S1:** Brain map of standardized effect sizes for hair cortisone associations with 34 regional (A) cortical volumes, (B) cortical thicknesses and (C) cortical surface areas, corrected for age, sex, study site, intracranial volume, imaging batch and imaging edits.


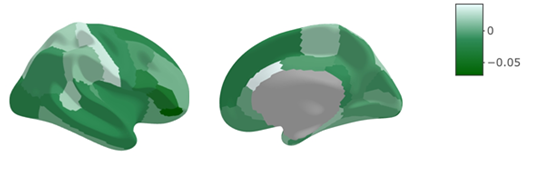

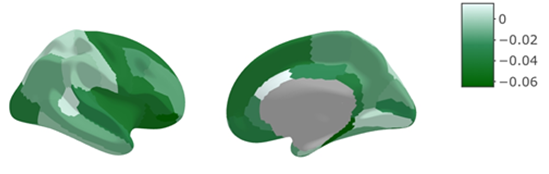


**A- Cortical Volume**

**B- Cortical Thickness**

**C- Cortical Surface Area**

β

β

β


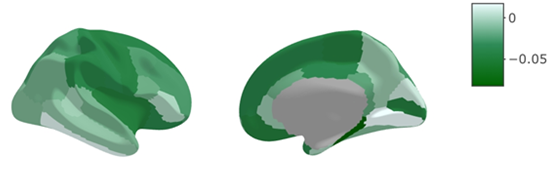


**Figure S2:** Brain map of standardized effect sizes for total glucocorticoid associations with 34 regional (A) cortical volumes, (B) cortical thicknesses and (C) cortical surface areas, corrected for age, sex, study site, intracranial volume, imaging batch and imaging edits.


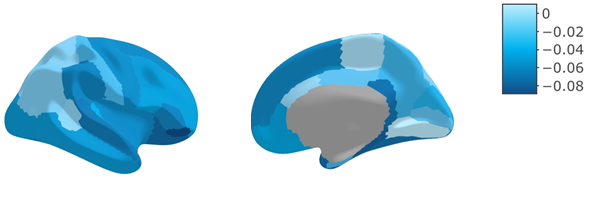

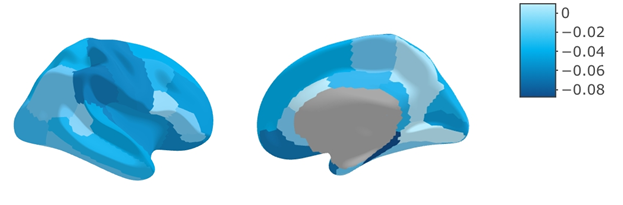

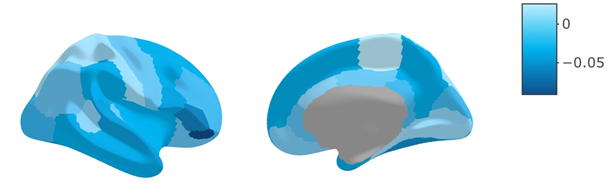


β

β

β

**A- Cortical Volume**

**C- Cortical Surface Area**

**B- Cortical**

**Thickness**

**Table S1:** Hair glucocorticoid associations with measures of (i) depression status and symptoms, (ii) current life stress (LTE sum score) and (iii) childhood trauma.

| **Measure** | **Log Cortisol (F)** | | | **Log Cortisone (E)** | | | **F+E Total** | | |
| --- | --- | --- | --- | --- | --- | --- | --- | --- | --- |
|  | **β** | **SE** | **pFDR** | **β** | **SE** | **pFDR** | **β** | **SE** | **pFDR** |
| **Depression Measures** |  |  |  |  |  |  |  |  |  |
| Lifetime MDD | 0.039 | 0.035 | 0.775 | 0.115 | 0.033 | **0.002** | 0.079 | 0.036 | 0.083 |
| Total QIDS | 0.010 | 0.036 | 0.775 | 0.089 | 0.034 | **0.014** | 0.064 | 0.037 | 0.130 |
| QIDS Severity | -0.016 | 0.036 | 0.775 | 0.071 | 0.034 | **0.038** | 0.042 | 0.037 | 0.257 |
| **Brief Life Events Questionnaire** |  |  |  |  |  |  |  |  |  |
| Total BLEQ* | -0.015 | 0.042 | 0.724 | 0.066 | 0.042 | 0.114 | -0.007 | 0.044 | 0.875 |
| **Childhood Trauma Questionnaire** |  |  |  |  |  |  |  |  |  |
| Total CTQ* | 0.048 | 0.036 | 0.187 | 0.083 | 0.034 | **0.017** | 0.076 | 0.037 | **0.040** |
| Physical Abuse | 0.070 | 0.036 | 0.290 | 0.062 | 0.035 | 0.149 | 0.068 | 0.037 | 0.163 |
| Sexual Abuse | 0.003 | 0.036 | 0.925 | 0.025 | 0.034 | 0.544 | 0.015 | 0.036 | 0.677 |
| Emotional Abuse | 0.056 | 0.035 | 0.340 | 0.087 | 0.034 | **0.034** | 0.089 | 0.037 | 0.094 |
| Physical Neglect | 0.015 | 0.036 | 0.825 | 0.090 | 0.035 | **0.034** | 0.048 | 0.037 | 0.302 |
| Emotional Neglect | 0.039 | 0.036 | 0.554 | 0.053 | 0.035 | 0.187 | 0.064 | 0.037 | 0.163 |
| Denial | -0.020 | 0.036 | 0.825 | -0.019 | 0.035 | 0.592 | -0.026 | 0.037 | 0.576 |

*Notes*: pFDR significant associations are marked in bold. Measures where no FDR correction was applied are marked with an asterisk. All models are adjusted for covariates- age, sex, study site and lab batch effects.

**Table S2**: Hair glucocorticoid associations with (i) global imaging phenotypes, (ii) lobar volumes, (iii) lobar thicknesses and (iv) lobar surface areas.

|  | **Structural Phenotype** | **Log Cortisol (F)** | | | **Log Cortisone (E)** | | | **F+E Total** | | |
| --- | --- | --- | --- | --- | --- | --- | --- | --- | --- | --- |
|  |  | **β** | **SE** | **pFDR** | **β** | **SE** | **pFDR** | **β** | **SE** | **pFDR** |
| **Global Brain Measures*** | Global Total Grey Matter | -0.057 | 0.019 | **0.003** | -0.023 | 0.019 | 0.214 | -0.053 | 0.020 | **0.009** |
|  | Global Cerebral White Matter | 0.001 | 0.025 | 0.965 | 0.020 | 0.025 | 0.417 | 0.014 | 0.026 | 0.593 |
|  | Global Cortical Volume | -0.104 | 0.030 | **0.001** | -0.059 | 0.029 | **0.043** | -0.103 | 0.032 | **0.001** |
|  | Global Cortical Thickness | -0.056 | 0.031 | 0.077 | -0.032 | 0.031 | 0.295 | -0.059 | 0.033 | 0.071 |
|  | Global Cortical Surface Area | -0.061 | 0.032 | 0.063 | -0.049 | 0.031 | 0.114 | -0.066 | 0.034 | **0.050** |
| **Lobar Volume** | Frontal Volume | -0.064 | 0.023 | **0.014** | -0.044 | 0.022 | 0.229 | -0.068 | 0.024 | **0.010** |
|  | Temporal Volume | -0.093 | 0.024 | **0.001** | -0.027 | 0.023 | 0.419 | -0.076 | 0.025 | **0.010** |
|  | Parietal Volume | -0.035 | 0.025 | 0.158 | -0.007 | 0.024 | 0.778 | -0.040 | 0.026 | 0.148 |
|  | Occipital Volume | -0.042 | 0.027 | 0.154 | -0.032 | 0.027 | 0.419 | -0.041 | 0.028 | 0.148 |
|  | Cingulate Volume | -0.064 | 0.028 | **0.040** | -0.026 | 0.028 | 0.429 | -0.059 | 0.029 | 0.076 |
| **Lobar Thickness** | Frontal Thickness | -0.043 | 0.033 | 0.315 | -0.036 | 0.032 | 0.645 | -0.051 | 0.034 | 0.328 |
|  | Temporal Thickness | -0.052 | 0.033 | 0.272 | -0.015 | 0.032 | 0.645 | -0.047 | 0.034 | 0.328 |
|  | Parietal Thickness | -0.019 | 0.030 | 0.662 | -0.019 | 0.028 | 0.645 | -0.034 | 0.031 | 0.328 |
|  | Occipital Thickness | -0.054 | 0.033 | 0.272 | -0.020 | 0.032 | 0.645 | -0.042 | 0.034 | 0.328 |
|  | Cingulate Thickness | -0.012 | 0.036 | 0.746 | -0.034 | 0.035 | 0.645 | -0.033 | 0.037 | 0.381 |
| **Lobar Surface Area** | Frontal Surface Area | -0.049 | 0.025 | 0.129 | -0.019 | 0.024 | 0.788 | -0.043 | 0.026 | 0.252 |
|  | Temporal Surface Area | -0.068 | 0.026 | **0.042** | -0.007 | 0.025 | 0.788 | -0.047 | 0.027 | 0.252 |
|  | Parietal Surface Area | -0.026 | 0.026 | 0.396 | 0.012 | 0.026 | 0.788 | -0.018 | 0.027 | 0.515 |
|  | Occipital Surface Area | -0.019 | 0.029 | 0.505 | -0.025 | 0.028 | 0.788 | -0.024 | 0.030 | 0.515 |
|  | Cingulate Surface Area | -0.035 | 0.028 | 0.342 | 0.010 | 0.027 | 0.788 | -0.019 | 0.029 | 0.515 |

*Notes*: pFDR significant associations are marked in bold. Measures where no FDR correction was applied are marked with an asterisk. All models are adjusted for covariates- age, sex, study site, lab batch effects, image edits, imaging batch and for the lobes intracranial volume.

**Table S3:** Hair cortisol associations with regional cortical metrics including volume, surface area and thickness.

| **Region** | **Cortical Volume** | | | **Cortical Surface Area** | | | **Cortical Thickness** | | |
| --- | --- | --- | --- | --- | --- | --- | --- | --- | --- |
|  | **Log Cortisol** | | | **Log Cortisol** | | | **Log Cortisol** | | |
|  | **β** | **SE** | **pFDR** | **β** | **SE** | **pFDR** | **β** | **SE** | **pFDR** |
| Bank Superior Temporal Sulcus | -0.035 | 0.029 | 0.345 | -0.030 | 0.030 | 0.531 | -0.021 | 0.030 | 0.693 |
| Caudal Anterior Cingulate | -0.019 | 0.026 | 0.582 | -0.028 | 0.027 | 0.531 | 0.031 | 0.030 | 0.606 |
| Caudal Middle Frontal | -0.035 | 0.029 | 0.345 | -0.033 | 0.030 | 0.531 | -0.016 | 0.031 | 0.746 |
| Cuneus | -0.042 | 0.030 | 0.274 | -0.021 | 0.030 | 0.715 | -0.062 | 0.031 | 0.503 |
| Entorhinal Cortex | -0.048 | 0.026 | 0.135 | -0.047 | 0.028 | 0.369 | -0.024 | 0.033 | 0.680 |
| Frontal Pole | -0.046 | 0.030 | 0.232 | -0.025 | 0.029 | 0.617 | -0.051 | 0.030 | 0.517 |
| Fusiform | -0.069 | 0.028 | 0.062 | -0.061 | 0.028 | 0.345 | -0.005 | 0.033 | 0.878 |
| Inferior Parietal | -0.012 | 0.028 | 0.740 | -0.017 | 0.028 | 0.761 | -0.004 | 0.029 | 0.878 |
| Inferior Temporal | -0.079 | 0.027 | 0.062 | -0.063 | 0.028 | 0.345 | -0.040 | 0.030 | 0.577 |
| Insula | -0.059 | 0.027 | 0.082 | -0.040 | 0.027 | 0.412 | -0.045 | 0.031 | 0.565 |
| Isthmus Cingulate | -0.075 | 0.029 | 0.062 | -0.029 | 0.028 | 0.531 | -0.027 | 0.031 | 0.606 |
| Lateral Occipital | -0.050 | 0.027 | 0.135 | -0.028 | 0.028 | 0.531 | -0.032 | 0.031 | 0.606 |
| Lateral Orbito Frontal | -0.062 | 0.026 | 0.062 | -0.017 | 0.029 | 0.761 | -0.061 | 0.033 | 0.503 |
| Lingual | -0.003 | 0.031 | 0.929 | 0.013 | 0.031 | 0.819 | -0.028 | 0.032 | 0.606 |
| Medial Orbito Frontal | -0.047 | 0.025 | 0.135 | -0.013 | 0.026 | 0.783 | -0.060 | 0.032 | 0.503 |
| Middle Temporal | -0.051 | 0.026 | 0.124 | -0.047 | 0.028 | 0.369 | -0.028 | 0.029 | 0.606 |
| Paracentral | 0.014 | 0.029 | 0.723 | 0.024 | 0.029 | 0.617 | 0.007 | 0.031 | 0.878 |
| Parahippocampal | -0.075 | 0.031 | 0.062 | -0.011 | 0.030 | 0.819 | -0.060 | 0.034 | 0.503 |
| Pars Opercularis | -0.038 | 0.028 | 0.279 | -0.032 | 0.029 | 0.531 | -0.007 | 0.030 | 0.878 |
| Pars Orbitalis | -0.072 | 0.027 | 0.062 | -0.071 | 0.028 | 0.345 | -0.038 | 0.031 | 0.577 |
| Pars Traingularis | -0.056 | 0.028 | 0.124 | -0.046 | 0.030 | 0.412 | -0.027 | 0.031 | 0.606 |
| Pericalcarine | -0.025 | 0.033 | 0.571 | -0.005 | 0.033 | 0.927 | -0.037 | 0.034 | 0.606 |
| Post Central | -0.016 | 0.028 | 0.669 | 0.002 | 0.028 | 0.936 | -0.028 | 0.030 | 0.606 |
| Posterior Cingulate | -0.002 | 0.028 | 0.929 | -0.002 | 0.027 | 0.936 | -0.020 | 0.031 | 0.693 |
| Precentral | -0.029 | 0.029 | 0.439 | -0.009 | 0.028 | 0.844 | -0.029 | 0.033 | 0.606 |
| Precuneus | -0.029 | 0.028 | 0.416 | -0.040 | 0.028 | 0.412 | 0.014 | 0.031 | 0.764 |
| Rostral Anterior Cingulate | -0.053 | 0.027 | 0.124 | -0.039 | 0.027 | 0.412 | 0.007 | 0.031 | 0.878 |
| Rostral Middle Frontal | -0.046 | 0.025 | 0.135 | -0.037 | 0.028 | 0.474 | -0.039 | 0.031 | 0.577 |
| Superior Frontal | -0.056 | 0.025 | 0.082 | -0.047 | 0.026 | 0.369 | -0.021 | 0.033 | 0.693 |
| Superior Parietal | -0.005 | 0.028 | 0.925 | 0.006 | 0.030 | 0.919 | -0.016 | 0.030 | 0.740 |
| Superior Temporal | -0.071 | 0.027 | 0.062 | -0.048 | 0.027 | 0.369 | -0.049 | 0.032 | 0.553 |
| Supramarginal | -0.068 | 0.027 | 0.062 | -0.052 | 0.027 | 0.369 | -0.046 | 0.030 | 0.553 |
| Temporal Pole | -0.096 | 0.030 | **0.049** | -0.056 | 0.029 | 0.369 | -0.084 | 0.033 | 0.346 |
| Transverse Temporal | -0.030 | 0.031 | 0.439 | -0.016 | 0.031 | 0.783 | -0.040 | 0.032 | 0.577 |

*Notes*: pFDR significant associations are marked in bold. All models are adjusted for covariates- age, sex, study site, lab batch effects, image edits, imaging batch, intracranial volume and hemisphere.

**Table S4:** Hair cortisone associations with regional cortical metrics including volume, surface area and thickness.

| **Region** | **Cortical Volume** | | | **Cortical Surface Area** | | | **Cortical Thickness** | | |
| --- | --- | --- | --- | --- | --- | --- | --- | --- | --- |
|  | **Log Cortisone** | | | **Log Cortisone** | | | **Log Cortisone** | | |
|  | **β** | **SE** | **pFDR** | **β** | **SE** | **pFDR** | **β** | **SE** | **pFDR** |
| Bank Superior Temporal Sulcus | 0.007 | 0.029 | 0.965 | 0.018 | 0.029 | 0.962 | -0.004 | 0.030 | 0.970 |
| Caudal Anterior Cingulate | 0.015 | 0.026 | 0.791 | 0.042 | 0.026 | 0.962 | -0.018 | 0.029 | 0.906 |
| Caudal Middle Frontal | -0.020 | 0.028 | 0.791 | -0.006 | 0.029 | 0.962 | -0.025 | 0.030 | 0.879 |
| Cuneus | -0.012 | 0.029 | 0.854 | -0.010 | 0.029 | 0.962 | 0.008 | 0.031 | 0.967 |
| Entorhinal Cortex | -0.002 | 0.027 | 0.965 | -0.008 | 0.028 | 0.962 | -0.002 | 0.032 | 0.987 |
| Frontal Pole | -0.018 | 0.029 | 0.791 | -0.002 | 0.028 | 0.962 | -0.015 | 0.030 | 0.906 |
| Fusiform | -0.016 | 0.027 | 0.791 | 0.005 | 0.028 | 0.962 | -0.013 | 0.032 | 0.909 |
| Inferior Parietal | -0.014 | 0.027 | 0.810 | -0.011 | 0.028 | 0.962 | -0.015 | 0.028 | 0.906 |
| Inferior Temporal | -0.005 | 0.026 | 0.965 | 0.004 | 0.028 | 0.962 | 0.014 | 0.030 | 0.906 |
| Insula | -0.047 | 0.026 | 0.489 | -0.018 | 0.026 | 0.962 | -0.051 | 0.030 | 0.559 |
| Isthmus Cingulate | -0.035 | 0.028 | 0.619 | -0.012 | 0.027 | 0.962 | -0.021 | 0.030 | 0.906 |
| Lateral Occipital | -0.039 | 0.026 | 0.531 | -0.023 | 0.027 | 0.962 | -0.012 | 0.030 | 0.909 |
| Lateral Orbito Frontal | -0.056 | 0.025 | 0.325 | -0.001 | 0.028 | 0.975 | -0.041 | 0.033 | 0.559 |
| Lingual | 0.003 | 0.030 | 0.965 | -0.005 | 0.030 | 0.962 | 0.018 | 0.032 | 0.906 |
| Medial Orbito Frontal | -0.035 | 0.025 | 0.539 | -0.007 | 0.026 | 0.962 | -0.050 | 0.031 | 0.559 |
| Middle Temporal | -0.014 | 0.025 | 0.791 | -0.020 | 0.027 | 0.962 | -0.008 | 0.029 | 0.967 |
| Paracentral | -0.016 | 0.028 | 0.791 | 0.016 | 0.028 | 0.962 | -0.048 | 0.030 | 0.559 |
| Parahippocampal | -0.065 | 0.030 | 0.325 | 0.017 | 0.028 | 0.962 | -0.083 | 0.033 | 0.425 |
| Pars Opercularis | -0.045 | 0.027 | 0.509 | -0.030 | 0.028 | 0.962 | -0.018 | 0.029 | 0.906 |
| Pars Orbitalis | -0.064 | 0.026 | 0.325 | -0.070 | 0.028 | 0.371 | -0.001 | 0.030 | 0.987 |
| Pars Traingularis | -0.045 | 0.028 | 0.509 | -0.046 | 0.030 | 0.962 | 0.000 | 0.030 | 0.987 |
| Pericalcarine | -0.027 | 0.032 | 0.791 | 0.003 | 0.032 | 0.962 | -0.048 | 0.033 | 0.559 |
| Post Central | -0.001 | 0.027 | 0.965 | 0.030 | 0.028 | 0.962 | -0.039 | 0.028 | 0.559 |
| Posterior Cingulate | -0.024 | 0.027 | 0.791 | -0.004 | 0.026 | 0.962 | -0.038 | 0.031 | 0.559 |
| Precentral | -0.042 | 0.028 | 0.531 | -0.013 | 0.027 | 0.962 | -0.045 | 0.032 | 0.559 |
| Precuneus | -0.016 | 0.027 | 0.791 | -0.007 | 0.028 | 0.962 | -0.005 | 0.030 | 0.970 |
| Rostral Anterior Cingulate | -0.021 | 0.027 | 0.791 | 0.003 | 0.027 | 0.962 | -0.006 | 0.030 | 0.970 |
| Rostral Middle Frontal | -0.017 | 0.025 | 0.791 | 0.004 | 0.027 | 0.962 | -0.026 | 0.030 | 0.879 |
| Superior Frontal | -0.043 | 0.024 | 0.489 | -0.021 | 0.025 | 0.962 | -0.043 | 0.032 | 0.559 |
| Superior Parietal | 0.002 | 0.027 | 0.965 | 0.018 | 0.029 | 0.962 | -0.027 | 0.029 | 0.870 |
| Superior Temporal | -0.032 | 0.027 | 0.619 | -0.020 | 0.027 | 0.962 | -0.016 | 0.031 | 0.906 |
| Supramarginal | -0.004 | 0.026 | 0.965 | 0.010 | 0.027 | 0.962 | -0.042 | 0.029 | 0.559 |
| Temporal Pole | -0.016 | 0.030 | 0.791 | 0.011 | 0.028 | 0.962 | -0.043 | 0.032 | 0.559 |
| Transverse Temporal | -0.036 | 0.030 | 0.619 | -0.005 | 0.030 | 0.962 | -0.051 | 0.032 | 0.559 |

*Notes:* All models are adjusted for covariates- age, sex, study site, lab batch, image edits, imaging batch, intracranial volume and hemisphere.

**Table S5:** Total glucocorticoid (F+E) associations with regional cortical metrics including volume, surface area and thickness.

| **Region** | **Cortical Volume** | | | **Cortical Surface Area** | | | **Cortical Thickness** | | |
| --- | --- | --- | --- | --- | --- | --- | --- | --- | --- |
|  | **F+E Total** | | | **F+E Total** | | | **F+E Total** | | |
|  | **β** | **SE** | **pFDR** | **β** | **SE** | **pFDR** | **β** | **SE** | **pFDR** |
| Bank Superior Temporal Sulcus | -0.010 | 0.030 | 0.798 | 0.000 | 0.031 | 0.990 | -0.013 | 0.031 | 0.775 |
| Caudal Anterior Cingulate | -0.005 | 0.027 | 0.877 | 0.002 | 0.028 | 0.980 | 0.009 | 0.031 | 0.827 |
| Caudal Middle Frontal | -0.049 | 0.030 | 0.191 | -0.035 | 0.031 | 0.637 | -0.036 | 0.033 | 0.473 |
| Cuneus | -0.037 | 0.031 | 0.348 | -0.025 | 0.031 | 0.719 | -0.038 | 0.033 | 0.473 |
| Entorhinal Cortex | -0.031 | 0.027 | 0.351 | -0.034 | 0.029 | 0.637 | -0.024 | 0.034 | 0.647 |
| Frontal Pole | -0.050 | 0.031 | 0.191 | -0.031 | 0.030 | 0.672 | -0.044 | 0.032 | 0.461 |
| Fusiform | -0.068 | 0.029 | 0.117 | -0.052 | 0.029 | 0.637 | -0.018 | 0.034 | 0.696 |
| Inferior Parietal | -0.015 | 0.029 | 0.700 | -0.013 | 0.029 | 0.890 | -0.023 | 0.030 | 0.647 |
| Inferior Temporal | -0.050 | 0.028 | 0.172 | -0.034 | 0.029 | 0.637 | -0.026 | 0.032 | 0.645 |
| Insula | -0.056 | 0.028 | 0.128 | -0.033 | 0.028 | 0.637 | -0.055 | 0.032 | 0.357 |
| Isthmus Cingulate | -0.068 | 0.030 | 0.117 | -0.023 | 0.029 | 0.719 | -0.036 | 0.032 | 0.473 |
| Lateral Occipital | -0.050 | 0.028 | 0.172 | -0.027 | 0.029 | 0.719 | -0.034 | 0.033 | 0.491 |
| Lateral Orbito Frontal | -0.073 | 0.027 | 0.111 | -0.014 | 0.030 | 0.890 | -0.060 | 0.035 | 0.357 |
| Lingual | 0.010 | 0.032 | 0.798 | 0.007 | 0.032 | 0.920 | 0.010 | 0.034 | 0.827 |
| Medial Orbito Frontal | -0.043 | 0.026 | 0.191 | -0.006 | 0.027 | 0.920 | -0.066 | 0.033 | 0.357 |
| Middle Temporal | -0.050 | 0.027 | 0.172 | -0.037 | 0.029 | 0.637 | -0.037 | 0.030 | 0.473 |
| Paracentral | 0.002 | 0.030 | 0.947 | 0.026 | 0.030 | 0.719 | -0.022 | 0.032 | 0.647 |
| Parahippocampal | -0.076 | 0.032 | 0.117 | 0.010 | 0.031 | 0.907 | -0.088 | 0.035 | 0.357 |
| Pars Opercularis | -0.046 | 0.029 | 0.191 | -0.041 | 0.030 | 0.637 | -0.007 | 0.031 | 0.848 |
| Pars Orbitalis | -0.089 | 0.028 | **0.050** | -0.091 | 0.029 | 0.068 | -0.022 | 0.032 | 0.647 |
| Pars Traingularis | -0.060 | 0.029 | 0.128 | -0.059 | 0.032 | 0.637 | -0.019 | 0.032 | 0.685 |
| Pericalcarine | -0.024 | 0.034 | 0.594 | 0.004 | 0.034 | 0.975 | -0.050 | 0.035 | 0.461 |
| Post Central | -0.030 | 0.029 | 0.401 | 0.008 | 0.029 | 0.907 | -0.059 | 0.031 | 0.357 |
| Posterior Cingulate | -0.024 | 0.029 | 0.499 | -0.010 | 0.028 | 0.890 | -0.034 | 0.033 | 0.491 |
| Precentral | -0.055 | 0.030 | 0.172 | -0.012 | 0.029 | 0.890 | -0.058 | 0.034 | 0.357 |
| Precuneus | -0.033 | 0.029 | 0.351 | -0.034 | 0.029 | 0.637 | 0.002 | 0.032 | 0.943 |
| Rostral Anterior Cingulate | -0.044 | 0.028 | 0.191 | -0.017 | 0.028 | 0.882 | -0.017 | 0.032 | 0.696 |
| Rostral Middle Frontal | -0.047 | 0.026 | 0.172 | -0.024 | 0.029 | 0.719 | -0.049 | 0.032 | 0.435 |
| Superior Frontal | -0.057 | 0.026 | 0.117 | -0.035 | 0.027 | 0.637 | -0.040 | 0.034 | 0.473 |
| Superior Parietal | -0.012 | 0.029 | 0.767 | 0.012 | 0.031 | 0.890 | -0.038 | 0.031 | 0.473 |
| Superior Temporal | -0.064 | 0.028 | 0.117 | -0.043 | 0.028 | 0.637 | -0.044 | 0.033 | 0.473 |
| Supramarginal | -0.059 | 0.028 | 0.125 | -0.035 | 0.028 | 0.637 | -0.064 | 0.031 | 0.357 |
| Temporal Pole | -0.070 | 0.031 | 0.117 | -0.038 | 0.030 | 0.637 | -0.069 | 0.034 | 0.357 |
| Transverse Temporal | -0.041 | 0.032 | 0.316 | -0.012 | 0.033 | 0.890 | -0.060 | 0.034 | 0.357 |

*Notes:* All models are adjusted for covariates- age, sex, study site, lab batch, image edits, imaging batch, intracranial volume and hemisphere.

**Table S6:** Hair glucocorticoid associations with subcortical volumes.

| **Subcortical Volume** | **Log Cortisol (F)** | | | **Log Cortisone (E)** | | | **F+E Total** | | |
| --- | --- | --- | --- | --- | --- | --- | --- | --- | --- |
|  | **β** | **SE** | **pFDR** | **β** | **SE** | **pFDR** | **β** | **SE** | **pFDR** |
| Nucleus Accumbens | -0.053 | 0.028 | 0.276 | -0.075 | 0.027 | **0.044** | -0.068 | 0.029 | 0.152 |
| Amygdala | -0.019 | 0.030 | 0.689 | -0.016 | 0.029 | 0.778 | -0.006 | 0.031 | 0.910 |
| Caudate | -0.023 | 0.032 | 0.689 | 0.004 | 0.031 | 0.899 | -0.021 | 0.034 | 0.855 |
| Hippocampus | -0.042 | 0.031 | 0.477 | -0.051 | 0.030 | 0.299 | -0.052 | 0.032 | 0.328 |
| Pallidum | 0.004 | 0.030 | 0.963 | 0.010 | 0.029 | 0.826 | 0.004 | 0.031 | 0.910 |
| Putamen | -0.022 | 0.031 | 0.689 | -0.033 | 0.030 | 0.549 | -0.030 | 0.032 | 0.688 |
| Thalamus | 0.047 | 0.026 | 0.276 | 0.039 | 0.025 | 0.299 | 0.041 | 0.027 | 0.328 |
| Ventral Diencephalon | -0.001 | 0.027 | 0.963 | -0.017 | 0.026 | 0.778 | -0.013 | 0.028 | 0.855 |

*Notes*: pFDR significant associations are marked in bold. All models are adjusted for covariates- age, sex, study site, lab batch effects, image edits, imaging batch, intracranical volume and hemisphere.

**Table S7:** Hair glucocorticoid associations with global DTI metrics.

|  | **DTI Measure** | **Log Cortisol (F)** | | | **Log Cortisone (E)** | | | **F+E Total** | | |
| --- | --- | --- | --- | --- | --- | --- | --- | --- | --- | --- |
|  |  | **β** | **SE** | **pFDR** | **β** | **SE** | **pFDR** | **β** | **SE** | **pFDR** |
| **Mean Diffusivity** | gMD* | 0.034 | 0.033 | 0.294 | 0.015 | 0.031 | 0.639 | 0.038 | 0.034 | 0.265 |
|  | Average MD* | 0.019 | 0.029 | 0.516 | 0.015 | 0.029 | 0.607 | 0.018 | 0.031 | 0.555 |
|  | Association Fibres | 0.035 | 0.033 | 0.725 | 0.012 | 0.032 | 0.702 | 0.037 | 0.034 | 0.569 |
|  | Commisural Fibres | 0.012 | 0.030 | 0.725 | 0.022 | 0.030 | 0.614 | 0.018 | 0.032 | 0.569 |
|  | Projection Fibres | 0.012 | 0.029 | 0.725 | 0.025 | 0.028 | 0.614 | 0.023 | 0.030 | 0.569 |
|  | Thalamic Radiations | 0.009 | 0.026 | 0.725 | 0.027 | 0.025 | 0.614 | 0.021 | 0.027 | 0.569 |
| **Fractional Anisotropy** | gFA* | -0.040 | 0.029 | 0.175 | -0.042 | 0.029 | 0.145 | -0.038 | 0.031 | 0.210 |
|  | Average FA* | -0.036 | 0.031 | 0.234 | -0.036 | 0.030 | 0.231 | -0.031 | 0.032 | 0.331 |
|  | Association Fibres | -0.038 | 0.030 | 0.429 | -0.031 | 0.029 | 0.293 | -0.037 | 0.031 | 0.494 |
|  | Commisural Fibres | -0.031 | 0.032 | 0.429 | -0.045 | 0.031 | 0.277 | -0.038 | 0.033 | 0.494 |
|  | Projection Fibres | -0.034 | 0.031 | 0.429 | -0.044 | 0.030 | 0.277 | -0.025 | 0.032 | 0.583 |
|  | Thalamic Radiations | -0.022 | 0.029 | 0.451 | -0.036 | 0.029 | 0.277 | -0.014 | 0.031 | 0.653 |

*Notes*: pFDR significant associations are marked in bold. Measures where no FDR correction was applied are marked with an asterisk. All models are adjusted for covariates- age, sex, study site and lab batch effects.

**Table S8:** Hair glucocorticoid associations with Mean Diffusivity of individual white matter tracts.

| **White Matter Tract** | **Mean Diffusivity** | | | | | | | | |
| --- | --- | --- | --- | --- | --- | --- | --- | --- | --- |
|  | **Log Cortisol (F)** | | | **Log Cortisone (E)** | | | **F+E Total** | | |
|  | **β** | **SE** | **pFDR** | **β** | **SE** | **pFDR** | **β** | **SE** | **pFDR** |
| Anterior Corona Radiata | -0.003 | 0.034 | 0.988 | 0.015 | 0.034 | 0.829 | 0.007 | 0.036 | 0.991 |
| Anterior Limb Internal Capsule | 0.015 | 0.032 | 0.988 | 0.025 | 0.032 | 0.776 | 0.020 | 0.034 | 0.897 |
| Cingulum (cingulate gyrus) | 0.013 | 0.029 | 0.988 | -0.032 | 0.028 | 0.776 | 0.000 | 0.030 | 0.991 |
| Cingulum (hippocampus) | 0.023 | 0.025 | 0.988 | 0.021 | 0.024 | 0.776 | 0.023 | 0.026 | 0.897 |
| Corona Radiata | 0.008 | 0.033 | 0.988 | 0.020 | 0.033 | 0.776 | 0.018 | 0.034 | 0.897 |
| Corticospinal Tract | 0.021 | 0.031 | 0.988 | -0.008 | 0.030 | 0.870 | 0.023 | 0.032 | 0.897 |
| External Capsule | 0.019 | 0.030 | 0.988 | 0.010 | 0.030 | 0.867 | 0.022 | 0.032 | 0.897 |
| Fornix(Cres)/ Stria Terminalis | 0.000 | 0.026 | 0.988 | 0.017 | 0.026 | 0.776 | 0.013 | 0.027 | 0.897 |
| Internal Capsule | 0.002 | 0.026 | 0.988 | 0.018 | 0.025 | 0.776 | 0.012 | 0.027 | 0.897 |
| Inferior- Fronto-Occipital Fasciculus | 0.044 | 0.030 | 0.988 | 0.054 | 0.029 | 0.776 | 0.050 | 0.031 | 0.897 |
| Posterior Corona Radiata | 0.014 | 0.030 | 0.988 | 0.019 | 0.030 | 0.776 | 0.020 | 0.031 | 0.897 |
| Posterior Limb of Internal Cpasule | -0.005 | 0.025 | 0.988 | 0.003 | 0.024 | 0.897 | 0.003 | 0.026 | 0.991 |
| Posterior Thalamic Radiation | 0.028 | 0.030 | 0.988 | 0.043 | 0.029 | 0.776 | 0.038 | 0.031 | 0.897 |
| Retrolenticular part of Internal Capsule | -0.004 | 0.024 | 0.988 | 0.022 | 0.024 | 0.776 | 0.009 | 0.025 | 0.922 |
| Superior Corona Radiata | 0.016 | 0.033 | 0.988 | 0.024 | 0.032 | 0.776 | 0.027 | 0.034 | 0.897 |
| Superior Fronto-Occipital Fasciculus | 0.015 | 0.033 | 0.988 | 0.017 | 0.032 | 0.800 | 0.006 | 0.034 | 0.991 |
| Superior Longitudinal Fasciculus | 0.011 | 0.030 | 0.988 | 0.020 | 0.030 | 0.776 | 0.013 | 0.032 | 0.897 |
| Sagittal Striatum | 0.043 | 0.029 | 0.988 | 0.040 | 0.029 | 0.776 | 0.051 | 0.031 | 0.897 |
| Uncinate Fasciculus | 0.025 | 0.031 | 0.988 | 0.020 | 0.033 | 0.776 | 0.029 | 0.032 | 0.897 |
| Body Corpus Callosum | -0.005 | 0.033 | 0.988 | 0.005 | 0.032 | 0.897 | 0.002 | 0.034 | 0.991 |
| Corpus Callosum | 0.009 | 0.031 | 0.988 | 0.020 | 0.030 | 0.776 | 0.016 | 0.032 | 0.897 |
| Fornix (column and body) | 0.033 | 0.034 | 0.988 | 0.010 | 0.032 | 0.867 | 0.035 | 0.035 | 0.897 |
| Genu Corpus Callosum | 0.020 | 0.031 | 0.988 | 0.023 | 0.031 | 0.776 | 0.022 | 0.033 | 0.897 |
| Splenium of Corpus Callosum | 0.016 | 0.029 | 0.988 | 0.034 | 0.028 | 0.776 | 0.025 | 0.030 | 0.897 |

*Notes*: All models are adjusted for covariates- age, sex, study site and lab batch effects.

**Table S9:** Hair glucocorticoid associations with Fractional Anisotropy of individual white matter tracts.

| **White Matter Tract** | **Fractional Anisotropy** | | | | | | | | | |
| --- | --- | --- | --- | --- | --- | --- | --- | --- | --- | --- |
|  | **Log Cortisol (F)** | | | **Log Cortisone (E)** | | | **F+E Total** | | | |
|  | **β** | **SE** | **pFDR** | **β** | **SE** | **pFDR** | **β** | **SE** | **pFDR** |  |
| Anterior Corona Radiata | -0.038 | 0.031 | 0.637 | -0.046 | 0.030 | 0.585 | -0.035 | 0.032 | 0.816 |  |
| Anterior Limb Internal Capsule | -0.021 | 0.033 | 0.820 | -0.025 | 0.033 | 0.594 | -0.018 | 0.034 | 0.837 |  |
| Cingulum (cingulate gyrus) | 0.007 | 0.032 | 0.902 | -0.023 | 0.031 | 0.594 | -0.005 | 0.033 | 0.942 |  |
| Cingulum (hippocampus) | -0.033 | 0.028 | 0.637 | -0.014 | 0.027 | 0.696 | -0.020 | 0.029 | 0.816 |  |
| Corona Radiata | -0.039 | 0.032 | 0.637 | -0.046 | 0.031 | 0.585 | -0.032 | 0.034 | 0.816 |  |
| Corticospinal Tract | -0.026 | 0.032 | 0.806 | -0.025 | 0.031 | 0.594 | -0.022 | 0.033 | 0.816 |  |
| External Capsule | -0.044 | 0.031 | 0.629 | -0.027 | 0.031 | 0.594 | -0.035 | 0.032 | 0.816 |  |
| Fornix(Cres)/ Stria Terminalis | -0.026 | 0.027 | 0.752 | -0.020 | 0.026 | 0.594 | -0.033 | 0.028 | 0.816 |  |
| Internal Capsule | -0.007 | 0.029 | 0.902 | -0.023 | 0.028 | 0.594 | 0.002 | 0.030 | 0.942 |  |
| Inferior- Fronto-Occipital Fasciculus | -0.019 | 0.032 | 0.820 | -0.017 | 0.031 | 0.696 | -0.021 | 0.033 | 0.816 |  |
| Posterior Corona Radiata | -0.036 | 0.033 | 0.637 | -0.041 | 0.032 | 0.594 | -0.031 | 0.034 | 0.816 |  |
| Posterior Limb of Internal Cpasule | -0.004 | 0.029 | 0.925 | -0.005 | 0.028 | 0.886 | 0.008 | 0.030 | 0.906 |  |
| Posterior Thalamic Radiation | -0.055 | 0.030 | 0.529 | -0.055 | 0.029 | 0.585 | -0.053 | 0.031 | 0.646 |  |
| Retrolenticular part of Internal Capsule | 0.007 | 0.028 | 0.902 | -0.031 | 0.027 | 0.594 | 0.012 | 0.029 | 0.867 |  |
| Superior Corona Radiata | -0.025 | 0.035 | 0.813 | -0.028 | 0.033 | 0.594 | -0.014 | 0.036 | 0.867 |  |
| Superior Fronto-Occipital Fasciculus | -0.078 | 0.032 | 0.360 | -0.022 | 0.031 | 0.594 | -0.070 | 0.033 | 0.509 |  |
| Superior Longitudinal Fasciculus | -0.050 | 0.033 | 0.627 | -0.047 | 0.032 | 0.585 | -0.030 | 0.034 | 0.816 |  |
| Sagittal Striatum | -0.049 | 0.030 | 0.578 | -0.043 | 0.029 | 0.585 | -0.050 | 0.031 | 0.646 |  |
| Uncinate Fasciculus | -0.010 | 0.033 | 0.902 | -0.032 | 0.033 | 0.594 | -0.022 | 0.034 | 0.816 |  |
| Body Corpus Callosum | -0.012 | 0.035 | 0.902 | -0.034 | 0.034 | 0.594 | -0.022 | 0.036 | 0.816 |  |
| Corpus Callosum | -0.025 | 0.032 | 0.806 | -0.039 | 0.031 | 0.594 | -0.031 | 0.033 | 0.816 |  |
| Fornix (column and body) | -0.015 | 0.032 | 0.902 | -0.004 | 0.031 | 0.890 | -0.012 | 0.033 | 0.867 |  |
| Genu Corpus Callosum | -0.060 | 0.032 | 0.529 | -0.067 | 0.031 | 0.585 | -0.067 | 0.033 | 0.509 |  |
| Splenium of Corpus Callosum | -0.002 | 0.028 | 0.936 | -0.007 | 0.027 | 0.862 | -0.002 | 0.029 | 0.942 |  |

*Notes*: All models are adjusted for covariates- age, sex, study site and lab batch effects.

**Table S10**: Hair glucocorticoid associations with (i) global imaging phenotypes, (ii) lobar volumes, (iii) lobar thicknesses and (iv) lobar surface areas in an unrelated subsample.

| **Structural Phenotype** | | **Log Cortisol (F)** | | | **Log Cortisone (E)** | | | **F+E Total** | | |
| --- | --- | --- | --- | --- | --- | --- | --- | --- | --- | --- |
|  |  | **β** | **SE** | **pFDR** | **β** | **SE** | **pFDR** | **β** | **SE** | **pFDR** |
| **Global Brain Measures*** | Global Total Grey Matter | -0.065 | 0.022 | **0.003** | -0.033 | 0.021 | 0.119 | -0.065 | 0.023 | **0.005** |
|  | Global Cerebral White Matter | 0.004 | 0.029 | 0.893 | 0.022 | 0.029 | 0.447 | 0.016 | 0.030 | 0.602 |
|  | Global Cortical Volume | -0.132 | 0.036 | **0.000** | -0.069 | 0.035 | **0.048** | -0.127 | 0.037 | **0.001** |
|  | Global Cortical Thickness | -0.070 | 0.037 | 0.057 | -0.038 | 0.036 | 0.287 | -0.070 | 0.039 | 0.071 |
|  | Global Cortical Surface Area | -0.070 | 0.038 | 0.066 | -0.056 | 0.037 | 0.124 | -0.082 | 0.039 | **0.038** |
| **Lobar Volume** | Frontal Volume | -0.079 | 0.027 | **0.008** | -0.045 | 0.026 | 0.389 | -0.083 | 0.028 | **0.014** |
|  | Temporal Volume | -0.093 | 0.027 | **0.003** | -0.032 | 0.027 | 0.389 | -0.078 | 0.028 | **0.014** |
|  | Parietal Volume | -0.034 | 0.028 | 0.222 | -0.007 | 0.027 | 0.795 | -0.043 | 0.029 | 0.136 |
|  | Occipital Volume | -0.049 | 0.032 | 0.150 | -0.027 | 0.031 | 0.466 | -0.055 | 0.033 | 0.121 |
|  | Cingulate Volume | -0.085 | 0.033 | **0.017** | -0.039 | 0.032 | 0.389 | -0.079 | 0.034 | **0.034** |
| **Lobar Thickness** | Frontal Thickness | -0.063 | 0.039 | 0.210 | -0.058 | 0.037 | 0.620 | -0.072 | 0.040 | 0.251 |
|  | Temporal Thickness | -0.061 | 0.038 | 0.210 | -0.018 | 0.037 | 0.748 | -0.054 | 0.039 | 0.251 |
|  | Parietal Thickness | -0.030 | 0.035 | 0.385 | -0.024 | 0.033 | 0.748 | -0.044 | 0.036 | 0.251 |
|  | Occipital Thickness | -0.058 | 0.038 | 0.210 | -0.012 | 0.037 | 0.748 | -0.045 | 0.039 | 0.251 |
|  | Cingulate Thickness | -0.050 | 0.042 | 0.298 | -0.035 | 0.041 | 0.748 | -0.059 | 0.044 | 0.251 |
| **Lobar Surface Area** | Frontal Surface Area | -0.050 | 0.029 | 0.211 | -0.011 | 0.028 | 0.866 | -0.046 | 0.030 | 0.324 |
|  | Temporal Surface Area | -0.059 | 0.029 | 0.193 | -0.013 | 0.028 | 0.866 | -0.046 | 0.030 | 0.324 |
|  | Parietal Surface Area | -0.015 | 0.029 | 0.616 | 0.017 | 0.029 | 0.866 | -0.013 | 0.031 | 0.662 |
|  | Occipital Surface Area | -0.021 | 0.034 | 0.616 | -0.024 | 0.033 | 0.866 | -0.033 | 0.035 | 0.562 |
|  | Cingulate Surface Area | -0.035 | 0.032 | 0.457 | 0.004 | 0.031 | 0.891 | -0.021 | 0.033 | 0.656 |

*Notes*: pFDR significant associations are marked in bold. Measures where no FDR correction was applied are marked with an asterisk. All models are adjusted for covariates- age, sex, study site, lab batch effects, image edits, imaging batch and for the lobes intracranial volume.

**Table S11**: Hair cortisol associations with regional cortical metrics including volume, surface area and thickness in an unrelated subsample.

| **Region** | **Cortical Volume** | | | **Cortical Surface Area** | | | **Cortical Thickness** | | |
| --- | --- | --- | --- | --- | --- | --- | --- | --- | --- |
|  | **Log Cortisol** | | | **Log Cortisol** | | | **Log Cortisol** | | |
|  | **β** | **SE** | **pFDR** | **β** | **SE** | **pFDR** | **β** | **SE** | **pFDR** |
| Bank Superior Temporal Sulcus | -0.037 | 0.034 | 0.424 | -0.023 | 0.034 | 0.727 | -0.041 | 0.035 | 0.535 |
| Caudal Anterior Cingulate | -0.024 | 0.031 | 0.596 | -0.018 | 0.032 | 0.739 | 0.013 | 0.036 | 0.820 |
| Caudal Middle Frontal | -0.068 | 0.035 | 0.118 | -0.056 | 0.035 | 0.572 | -0.025 | 0.037 | 0.664 |
| Cuneus | -0.036 | 0.034 | 0.448 | -0.002 | 0.035 | 0.962 | -0.054 | 0.037 | 0.509 |
| Entorhinal Cortex | -0.060 | 0.031 | 0.118 | -0.046 | 0.033 | 0.572 | -0.042 | 0.038 | 0.535 |
| Frontal Pole | -0.056 | 0.035 | 0.206 | -0.028 | 0.032 | 0.727 | -0.070 | 0.035 | 0.509 |
| Fusiform | -0.071 | 0.032 | 0.089 | -0.054 | 0.032 | 0.572 | -0.009 | 0.039 | 0.871 |
| Inferior Parietal | -0.022 | 0.032 | 0.618 | -0.022 | 0.032 | 0.727 | -0.004 | 0.034 | 0.934 |
| Inferior Temporal | -0.062 | 0.032 | 0.118 | -0.050 | 0.033 | 0.572 | -0.026 | 0.035 | 0.660 |
| Insula | -0.082 | 0.030 | 0.060 | -0.054 | 0.031 | 0.572 | -0.056 | 0.037 | 0.509 |
| Isthmus Cingulate | -0.082 | 0.033 | 0.060 | -0.024 | 0.031 | 0.727 | -0.063 | 0.036 | 0.509 |
| Lateral Occipital | -0.057 | 0.032 | 0.152 | -0.039 | 0.032 | 0.572 | -0.027 | 0.036 | 0.660 |
| Lateral Orbito Frontal | -0.076 | 0.030 | 0.060 | -0.026 | 0.033 | 0.727 | -0.057 | 0.039 | 0.509 |
| Lingual | -0.012 | 0.036 | 0.791 | 0.009 | 0.036 | 0.936 | -0.027 | 0.038 | 0.660 |
| Medial Orbito Frontal | -0.054 | 0.029 | 0.137 | -0.023 | 0.030 | 0.727 | -0.056 | 0.037 | 0.509 |
| Middle Temporal | -0.042 | 0.030 | 0.280 | -0.037 | 0.031 | 0.572 | -0.012 | 0.034 | 0.820 |
| Paracentral | -0.015 | 0.033 | 0.725 | 0.024 | 0.033 | 0.727 | -0.017 | 0.036 | 0.774 |
| Parahippocampal | -0.073 | 0.036 | 0.118 | -0.002 | 0.034 | 0.962 | -0.080 | 0.039 | 0.509 |
| Pars Opercularis | -0.024 | 0.033 | 0.618 | -0.013 | 0.034 | 0.893 | -0.024 | 0.035 | 0.660 |
| Pars Orbitalis | -0.078 | 0.031 | 0.060 | -0.079 | 0.033 | 0.560 | -0.022 | 0.036 | 0.685 |
| Pars Traingularis | -0.043 | 0.033 | 0.323 | -0.028 | 0.035 | 0.727 | -0.029 | 0.036 | 0.660 |
| Pericalcarine | -0.006 | 0.038 | 0.867 | 0.026 | 0.038 | 0.727 | -0.042 | 0.040 | 0.543 |
| Post Central | -0.019 | 0.033 | 0.668 | 0.020 | 0.032 | 0.736 | -0.042 | 0.035 | 0.535 |
| Posterior Cingulate | -0.014 | 0.032 | 0.725 | 0.003 | 0.031 | 0.962 | -0.042 | 0.037 | 0.535 |
| Precentral | -0.041 | 0.034 | 0.377 | 0.005 | 0.033 | 0.961 | -0.048 | 0.039 | 0.535 |
| Precuneus | -0.018 | 0.032 | 0.668 | -0.021 | 0.032 | 0.727 | 0.002 | 0.036 | 0.957 |
| Rostral Anterior Cingulate | -0.078 | 0.032 | 0.060 | -0.046 | 0.032 | 0.572 | -0.009 | 0.036 | 0.871 |
| Rostral Middle Frontal | -0.078 | 0.030 | 0.060 | -0.051 | 0.033 | 0.572 | -0.053 | 0.037 | 0.509 |
| Superior Frontal | -0.067 | 0.029 | 0.084 | -0.038 | 0.030 | 0.572 | -0.037 | 0.039 | 0.601 |
| Superior Parietal | -0.009 | 0.032 | 0.812 | 0.008 | 0.034 | 0.936 | -0.025 | 0.035 | 0.660 |
| Superior Temporal | -0.093 | 0.031 | 0.060 | -0.050 | 0.031 | 0.572 | -0.066 | 0.038 | 0.509 |
| Supramarginal | -0.061 | 0.031 | 0.118 | -0.038 | 0.031 | 0.572 | -0.042 | 0.035 | 0.535 |
| Temporal Pole | -0.093 | 0.036 | 0.060 | -0.045 | 0.033 | 0.572 | -0.075 | 0.039 | 0.509 |
| Transverse Temporal | -0.029 | 0.036 | 0.595 | -0.008 | 0.036 | 0.936 | -0.044 | 0.038 | 0.535 |

*Notes:* All models are adjusted for covariates- age, sex, study site, lab batch, image edits, imaging batch, intracranial volume and hemisphere.

**Table S12**: Hair cortisone associations with regional cortical metrics including volume, surface area and thickness in an unrelated subsample.

| **Region** | **Cortical Volume** | | | **Cortical Surface Area** | | | **Cortical Thickness** | | |
| --- | --- | --- | --- | --- | --- | --- | --- | --- | --- |
|  | **Log Cortisone** | | | **Log Cortisone** | | | **Log Cortisone** | | |
|  | **β** | **SE** | **pFDR** | **β** | **SE** | **pFDR** | **β** | **SE** | **pFDR** |
| Bank Superior Temporal Sulcus | 0.010 | 0.034 | 0.931 | 0.014 | 0.034 | 0.959 | -0.008 | 0.035 | 0.929 |
| Caudal Anterior Cingulate | 0.007 | 0.030 | 0.931 | 0.038 | 0.031 | 0.959 | -0.010 | 0.034 | 0.929 |
| Caudal Middle Frontal | -0.029 | 0.034 | 0.923 | -0.007 | 0.034 | 0.959 | -0.023 | 0.035 | 0.776 |
| Cuneus | 0.003 | 0.034 | 0.969 | 0.009 | 0.034 | 0.959 | 0.026 | 0.036 | 0.776 |
| Entorhinal Cortex | -0.019 | 0.031 | 0.931 | -0.013 | 0.033 | 0.959 | -0.024 | 0.038 | 0.776 |
| Frontal Pole | -0.001 | 0.034 | 0.973 | 0.018 | 0.032 | 0.959 | -0.033 | 0.035 | 0.759 |
| Fusiform | -0.026 | 0.031 | 0.923 | -0.009 | 0.031 | 0.959 | -0.002 | 0.038 | 0.969 |
| Inferior Parietal | -0.014 | 0.031 | 0.931 | -0.014 | 0.032 | 0.959 | -0.018 | 0.033 | 0.791 |
| Inferior Temporal | 0.014 | 0.031 | 0.931 | 0.014 | 0.032 | 0.959 | 0.023 | 0.035 | 0.776 |
| Insula | -0.067 | 0.029 | 0.383 | -0.046 | 0.030 | 0.959 | -0.056 | 0.036 | 0.687 |
| Isthmus Cingulate | -0.011 | 0.033 | 0.931 | 0.014 | 0.031 | 0.959 | -0.025 | 0.035 | 0.776 |
| Lateral Occipital | -0.024 | 0.031 | 0.923 | -0.017 | 0.032 | 0.959 | -0.009 | 0.036 | 0.929 |
| Lateral Orbito Frontal | -0.049 | 0.030 | 0.400 | 0.003 | 0.033 | 0.963 | -0.041 | 0.038 | 0.751 |
| Lingual | -0.011 | 0.035 | 0.931 | -0.024 | 0.035 | 0.959 | 0.021 | 0.037 | 0.791 |
| Medial Orbito Frontal | -0.020 | 0.029 | 0.931 | -0.005 | 0.030 | 0.963 | -0.033 | 0.037 | 0.759 |
| Middle Temporal | -0.025 | 0.029 | 0.923 | -0.031 | 0.031 | 0.959 | 0.005 | 0.034 | 0.956 |
| Paracentral | -0.059 | 0.033 | 0.395 | -0.007 | 0.032 | 0.959 | -0.059 | 0.034 | 0.687 |
| Parahippocampal | -0.074 | 0.035 | 0.395 | -0.002 | 0.033 | 0.963 | -0.077 | 0.039 | 0.687 |
| Pars Opercularis | -0.037 | 0.032 | 0.847 | -0.020 | 0.033 | 0.959 | -0.030 | 0.033 | 0.759 |
| Pars Orbitalis | -0.081 | 0.031 | 0.288 | -0.081 | 0.032 | 0.400 | -0.001 | 0.035 | 0.969 |
| Pars Traingularis | -0.032 | 0.033 | 0.912 | -0.026 | 0.034 | 0.959 | -0.016 | 0.035 | 0.852 |
| Pericalcarine | -0.006 | 0.037 | 0.931 | 0.025 | 0.037 | 0.959 | -0.035 | 0.038 | 0.759 |
| Post Central | 0.005 | 0.032 | 0.931 | 0.048 | 0.032 | 0.959 | -0.040 | 0.033 | 0.751 |
| Posterior Cingulate | -0.035 | 0.032 | 0.847 | -0.006 | 0.030 | 0.959 | -0.042 | 0.036 | 0.751 |
| Precentral | -0.056 | 0.033 | 0.395 | 0.009 | 0.032 | 0.959 | -0.077 | 0.038 | 0.687 |
| Precuneus | -0.008 | 0.031 | 0.931 | 0.002 | 0.032 | 0.963 | -0.003 | 0.035 | 0.969 |
| Rostral Anterior Cingulate | -0.053 | 0.031 | 0.395 | -0.017 | 0.031 | 0.959 | -0.009 | 0.036 | 0.929 |
| Rostral Middle Frontal | -0.019 | 0.029 | 0.931 | 0.015 | 0.032 | 0.959 | -0.045 | 0.036 | 0.751 |
| Superior Frontal | -0.048 | 0.028 | 0.395 | -0.013 | 0.030 | 0.959 | -0.058 | 0.037 | 0.687 |
| Superior Parietal | 0.006 | 0.031 | 0.931 | 0.030 | 0.033 | 0.959 | -0.027 | 0.034 | 0.776 |
| Superior Temporal | -0.054 | 0.031 | 0.395 | -0.024 | 0.031 | 0.959 | -0.039 | 0.037 | 0.751 |
| Supramarginal | -0.011 | 0.031 | 0.931 | 0.011 | 0.031 | 0.959 | -0.042 | 0.034 | 0.751 |
| Temporal Pole | -0.020 | 0.035 | 0.931 | 0.011 | 0.032 | 0.959 | -0.058 | 0.038 | 0.687 |
| Transverse Temporal | -0.019 | 0.035 | 0.931 | 0.018 | 0.035 | 0.959 | -0.054 | 0.037 | 0.717 |

*Notes:* All models are adjusted for covariates- age, sex, study site, lab batch, image edits, imaging batch, intracranial volume and hemisphere.

**Table S13**: Total hair glucocorticoid (F+E) associations with regional cortical metrics including volume, surface area and thickness in an unrelated subsample.

| **Region** | **Cortical Volume** | | | **Cortical Surface Area** | | | **Cortical Thickness** | | |
| --- | --- | --- | --- | --- | --- | --- | --- | --- | --- |
|  | **F+E Total** | | | **F+E Total** | | | **F+E Total** | | |
|  | **β** | **SE** | **pFDR** | **β** | **SE** | **pFDR** | **β** | **SE** | **pFDR** |
| Bank Superior Temporal Sulcus | -0.012 | 0.035 | 0.801 | 0.004 | 0.036 | 0.973 | -0.029 | 0.036 | 0.614 |
| Caudal Anterior Cingulate | -0.009 | 0.032 | 0.805 | 0.009 | 0.033 | 0.973 | 0.004 | 0.037 | 0.915 |
| Caudal Middle Frontal | -0.067 | 0.036 | 0.173 | -0.048 | 0.036 | 0.813 | -0.032 | 0.038 | 0.614 |
| Cuneus | -0.036 | 0.036 | 0.436 | -0.014 | 0.036 | 0.938 | -0.028 | 0.038 | 0.614 |
| Entorhinal Cortex | -0.043 | 0.032 | 0.298 | -0.032 | 0.034 | 0.901 | -0.042 | 0.039 | 0.509 |
| Frontal Pole | -0.051 | 0.036 | 0.296 | -0.027 | 0.033 | 0.930 | -0.062 | 0.037 | 0.394 |
| Fusiform | -0.073 | 0.033 | 0.117 | -0.054 | 0.033 | 0.813 | -0.017 | 0.040 | 0.786 |
| Inferior Parietal | -0.023 | 0.033 | 0.594 | -0.017 | 0.033 | 0.930 | -0.024 | 0.036 | 0.614 |
| Inferior Temporal | -0.032 | 0.033 | 0.436 | -0.023 | 0.034 | 0.930 | -0.012 | 0.037 | 0.822 |
| Insula | -0.086 | 0.031 | 0.056 | -0.063 | 0.032 | 0.813 | -0.060 | 0.038 | 0.394 |
| Isthmus Cingulate | -0.056 | 0.035 | 0.240 | -0.005 | 0.032 | 0.973 | -0.055 | 0.037 | 0.394 |
| Lateral Occipital | -0.061 | 0.033 | 0.173 | -0.039 | 0.034 | 0.813 | -0.034 | 0.038 | 0.614 |
| Lateral Orbito Frontal | -0.082 | 0.031 | 0.056 | -0.025 | 0.035 | 0.930 | -0.052 | 0.040 | 0.428 |
| Lingual | -0.004 | 0.037 | 0.923 | -0.004 | 0.037 | 0.973 | 0.013 | 0.039 | 0.822 |
| Medial Orbito Frontal | -0.044 | 0.030 | 0.289 | -0.015 | 0.031 | 0.930 | -0.050 | 0.039 | 0.428 |
| Middle Temporal | -0.047 | 0.031 | 0.264 | -0.035 | 0.033 | 0.813 | -0.025 | 0.035 | 0.614 |
| Paracentral | -0.046 | 0.034 | 0.298 | 0.002 | 0.034 | 0.973 | -0.044 | 0.037 | 0.441 |
| Parahippocampal | -0.080 | 0.037 | 0.121 | 0.002 | 0.035 | 0.973 | -0.099 | 0.040 | 0.394 |
| Pars Opercularis | -0.027 | 0.034 | 0.548 | -0.016 | 0.035 | 0.930 | -0.024 | 0.036 | 0.614 |
| Pars Orbitalis | -0.106 | 0.032 | **0.037** | -0.109 | 0.034 | 0.048 | -0.007 | 0.037 | 0.895 |
| Pars Traingularis | -0.047 | 0.034 | 0.298 | -0.040 | 0.036 | 0.813 | -0.026 | 0.037 | 0.614 |
| Pericalcarine | -0.011 | 0.039 | 0.805 | 0.025 | 0.039 | 0.930 | -0.053 | 0.041 | 0.428 |
| Post Central | -0.033 | 0.034 | 0.436 | 0.020 | 0.033 | 0.930 | -0.068 | 0.036 | 0.394 |
| Posterior Cingulate | -0.037 | 0.033 | 0.416 | -0.006 | 0.032 | 0.973 | -0.056 | 0.038 | 0.394 |
| Precentral | -0.070 | 0.035 | 0.145 | 0.004 | 0.034 | 0.973 | -0.084 | 0.040 | 0.394 |
| Precuneus | -0.020 | 0.033 | 0.642 | -0.015 | 0.033 | 0.930 | -0.006 | 0.038 | 0.895 |
| Rostral Anterior Cingulate | -0.084 | 0.033 | 0.056 | -0.042 | 0.033 | 0.813 | -0.027 | 0.038 | 0.614 |
| Rostral Middle Frontal | -0.078 | 0.031 | 0.056 | -0.039 | 0.034 | 0.813 | -0.066 | 0.038 | 0.394 |
| Superior Frontal | -0.078 | 0.030 | 0.056 | -0.037 | 0.031 | 0.813 | -0.058 | 0.040 | 0.394 |
| Superior Parietal | -0.014 | 0.033 | 0.766 | 0.020 | 0.035 | 0.930 | -0.043 | 0.036 | 0.441 |
| Superior Temporal | -0.091 | 0.033 | 0.056 | -0.050 | 0.032 | 0.813 | -0.065 | 0.039 | 0.394 |
| Supramarginal | -0.065 | 0.032 | 0.141 | -0.034 | 0.032 | 0.813 | -0.061 | 0.036 | 0.394 |
| Temporal Pole | -0.061 | 0.037 | 0.240 | -0.028 | 0.034 | 0.930 | -0.062 | 0.040 | 0.394 |
| Transverse Temporal | -0.037 | 0.038 | 0.436 | 0.001 | 0.038 | 0.975 | -0.067 | 0.039 | 0.394 |

*Notes:* All models are adjusted for covariates- age, sex, study site, lab batch, image edits, imaging batch, intracranial volume and hemisphere.

**Table S14**: Hair glucocorticoid associations with subcortical volumes in an unrelated subsample.

| **Subcortical Volume** | **Log Cortisol (F)** | | | **Log Cortisone (E)** | | | **F+E Total** | | |
| --- | --- | --- | --- | --- | --- | --- | --- | --- | --- |
|  | **β** | **SE** | **pFDR** | **β** | **SE** | **pFDR** | **β** | **SE** | **pFDR** |
| Nucleus Accumbens | -0.045 | 0.033 | 0.680 | -0.057 | 0.033 | 0.331 | -0.056 | 0.034 | 0.398 |
| Amygdala | -0.013 | 0.034 | 0.797 | 0.000 | 0.034 | 0.998 | 0.012 | 0.036 | 0.807 |
| Caudate | -0.023 | 0.038 | 0.797 | 0.001 | 0.037 | 0.998 | -0.020 | 0.039 | 0.807 |
| Hippocampus | -0.035 | 0.035 | 0.797 | -0.050 | 0.034 | 0.383 | -0.048 | 0.037 | 0.509 |
| Pallidum | 0.017 | 0.035 | 0.797 | 0.002 | 0.034 | 0.998 | 0.012 | 0.036 | 0.807 |
| Putamen | -0.004 | 0.036 | 0.921 | -0.023 | 0.035 | 0.998 | -0.009 | 0.037 | 0.807 |
| Thalamus | 0.058 | 0.029 | 0.381 | 0.052 | 0.028 | 0.331 | 0.058 | 0.030 | 0.398 |
| Ventral Diencephalon | 0.024 | 0.032 | 0.797 | -0.005 | 0.031 | 0.998 | 0.014 | 0.033 | 0.807 |

*Notes:* All models are adjusted for covariates- age, sex, study site, lab batch, image edits, imaging batch, intracranial volume and hemisphere.

**Table S15**: Hair glucocorticoid associations with global DTI metrics in an unrelated subsample.

|  | **DTI Measure** | **Log Cortisol (F)** | | | **Log Cortisone (E)** | | | **F+E Total** | | |
| --- | --- | --- | --- | --- | --- | --- | --- | --- | --- | --- |
|  |  | **β** | **SE** | **pFDR** | **β** | **SE** | **pFDR** | **β** | **SE** | **pFDR** |
| **Mean Diffusivity** | gMD* | 0.002 | 0.039 | 0.953 | 0.010 | 0.037 | 0.788 | 0.016 | 0.040 | 0.685 |
|  | Average MD* | 0.014 | 0.035 | 0.692 | 0.007 | 0.034 | 0.838 | 0.011 | 0.036 | 0.762 |
|  | Association Fibres | 0.000 | 0.039 | 0.995 | 0.006 | 0.037 | 0.865 | 0.014 | 0.040 | 0.755 |
|  | Commisural Fibres | 0.005 | 0.036 | 0.995 | 0.025 | 0.035 | 0.639 | 0.012 | 0.037 | 0.755 |
|  | Projection Fibres | 0.018 | 0.034 | 0.995 | 0.028 | 0.034 | 0.639 | 0.032 | 0.036 | 0.734 |
|  | Thalamic Radiations | 0.017 | 0.031 | 0.995 | 0.024 | 0.031 | 0.639 | 0.029 | 0.033 | 0.734 |
| **Fractional Anisotropy** | gFA* | -0.049 | 0.036 | 0.166 | -0.051 | 0.035 | 0.141 | -0.052 | 0.037 | 0.158 |
|  | Average FA* | -0.047 | 0.037 | 0.205 | -0.045 | 0.036 | 0.209 | -0.045 | 0.038 | 0.240 |
|  | Association Fibres | -0.046 | 0.036 | 0.322 | -0.037 | 0.035 | 0.298 | -0.049 | 0.038 | 0.317 |
|  | Commisural Fibres | -0.026 | 0.037 | 0.480 | -0.054 | 0.037 | 0.231 | -0.039 | 0.039 | 0.317 |
|  | Projection Fibres | -0.049 | 0.037 | 0.322 | -0.053 | 0.036 | 0.231 | -0.043 | 0.038 | 0.317 |
|  | Thalamic Radiations | -0.041 | 0.035 | 0.322 | -0.047 | 0.034 | 0.231 | -0.037 | 0.037 | 0.317 |

*Notes*: pFDR significant associations are marked in bold. Measures where no FDR correction was applied are marked with an asterisk. All models are adjusted for covariates- age, sex, study site and lab batch effects.

**Table S16:** Hair glucocorticoid associations with Mean Diffusivity of individual white matter tracts in an unrelated subsample.

| **White Matter Tract** | **Mean Diffusivity** | | | | | | | | | |
| --- | --- | --- | --- | --- | --- | --- | --- | --- | --- | --- |
|  | **Log Cortisol (F)** | | | **Log Cortisone (E)** | | | **F+E Total** | | | |
|  | **β** | **SE** | **pFDR** | **β** | **SE** | **pFDR** | **β** | **SE** | **pFDR** |  |
| Anterior Corona Radiata | -0.008 | 0.040 | 0.982 | 0.020 | 0.040 | 0.913 | 0.008 | 0.042 | 0.868 |  |
| Anterior Limb Internal Capsule | 0.011 | 0.038 | 0.982 | 0.015 | 0.038 | 0.914 | 0.022 | 0.040 | 0.868 |  |
| Cingulum (cingulate gyrus) | -0.002 | 0.034 | 0.982 | -0.028 | 0.033 | 0.913 | -0.008 | 0.036 | 0.868 |  |
| Cingulum (hippocampus) | 0.036 | 0.029 | 0.982 | 0.025 | 0.028 | 0.913 | 0.032 | 0.030 | 0.868 |  |
| Corona Radiata | 0.009 | 0.039 | 0.982 | 0.029 | 0.039 | 0.913 | 0.026 | 0.041 | 0.868 |  |
| Corticospinal Tract | 0.023 | 0.036 | 0.982 | -0.005 | 0.036 | 0.932 | 0.023 | 0.037 | 0.868 |  |
| External Capsule | 0.017 | 0.036 | 0.982 | 0.011 | 0.036 | 0.914 | 0.026 | 0.038 | 0.868 |  |
| Fornix(Cres)/ Stria Terminalis | -0.010 | 0.031 | 0.982 | -0.009 | 0.030 | 0.914 | -0.005 | 0.032 | 0.868 |  |
| Internal Capsule | 0.010 | 0.031 | 0.982 | 0.011 | 0.030 | 0.914 | 0.020 | 0.032 | 0.868 |  |
| Inferior- Fronto-Occipital Fasciculus | 0.045 | 0.035 | 0.982 | 0.062 | 0.034 | 0.913 | 0.056 | 0.036 | 0.868 |  |
| Posterior Corona Radiata | 0.022 | 0.036 | 0.982 | 0.035 | 0.035 | 0.913 | 0.038 | 0.037 | 0.868 |  |
| Posterior Limb of Internal Cpasule | 0.010 | 0.030 | 0.982 | -0.007 | 0.029 | 0.914 | 0.012 | 0.031 | 0.868 |  |
| Posterior Thalamic Radiation | 0.030 | 0.035 | 0.982 | 0.054 | 0.035 | 0.913 | 0.045 | 0.036 | 0.868 |  |
| Retrolenticular part of Internal Capsule | 0.003 | 0.029 | 0.982 | 0.023 | 0.028 | 0.913 | 0.016 | 0.030 | 0.868 |  |
| Superior Corona Radiata | 0.018 | 0.039 | 0.982 | 0.031 | 0.038 | 0.913 | 0.035 | 0.040 | 0.868 |  |
| Superior Fronto-Occipital Fasciculus | 0.021 | 0.039 | 0.982 | 0.026 | 0.038 | 0.913 | 0.024 | 0.040 | 0.868 |  |
| Superior Longitudinal Fasciculus | 0.017 | 0.036 | 0.982 | 0.021 | 0.035 | 0.913 | 0.022 | 0.037 | 0.868 |  |
| Sagittal Striatum | 0.056 | 0.035 | 0.982 | 0.050 | 0.034 | 0.913 | 0.068 | 0.036 | 0.868 |  |
| Uncinate Fasciculus | 0.025 | 0.037 | 0.982 | 0.022 | 0.038 | 0.913 | 0.030 | 0.038 | 0.868 |  |
| Body Corpus Callosum | -0.014 | 0.038 | 0.982 | -0.003 | 0.038 | 0.932 | -0.013 | 0.040 | 0.868 |  |
| Corpus Callosum | 0.001 | 0.036 | 0.982 | 0.020 | 0.036 | 0.913 | 0.007 | 0.037 | 0.868 |  |
| Fornix (column and body) | -0.004 | 0.039 | 0.982 | 0.003 | 0.038 | 0.932 | 0.010 | 0.041 | 0.868 |  |
| Genu Corpus Callosum | 0.017 | 0.037 | 0.982 | 0.040 | 0.037 | 0.913 | 0.026 | 0.038 | 0.868 |  |
| Splenium of Corpus Callosum | 0.008 | 0.034 | 0.982 | 0.028 | 0.033 | 0.913 | 0.015 | 0.035 | 0.868 |  |

*Notes*: All models are adjusted for covariates- age, sex, study site and lab batch effects.

**Table S17:** Hair glucocorticoid associations with Fractional Anisotropy of individual white matter tracts in an unrelated subsample.

| **White Matter Tract** | **Fractional Anisotropy** | | | | | | | | |
| --- | --- | --- | --- | --- | --- | --- | --- | --- | --- |
|  | **Log Cortisol (F)** | | | **Log Cortisone (E)** | | | **F+E Total** | | |
|  | **β** | **SE** | **pFDR** | **β** | **SE** | **pFDR** | **β** | **SE** | **pFDR** |
| Anterior Corona Radiata | -0.034 | 0.037 | 0.788 | -0.064 | 0.036 | 0.469 | -0.035 | 0.038 | 0.727 |
| Anterior Limb Internal Capsule | -0.024 | 0.039 | 0.847 | -0.038 | 0.039 | 0.645 | -0.037 | 0.041 | 0.727 |
| Cingulum (cingulate gyrus) | -0.019 | 0.038 | 0.847 | -0.031 | 0.038 | 0.645 | -0.023 | 0.039 | 0.848 |
| Cingulum (hippocampus) | -0.065 | 0.033 | 0.311 | -0.026 | 0.032 | 0.645 | -0.048 | 0.035 | 0.585 |
| Corona Radiata | -0.037 | 0.038 | 0.788 | -0.054 | 0.038 | 0.539 | -0.031 | 0.040 | 0.748 |
| Corticospinal Tract | -0.045 | 0.037 | 0.612 | -0.022 | 0.037 | 0.743 | -0.039 | 0.039 | 0.727 |
| External Capsule | -0.053 | 0.037 | 0.528 | -0.045 | 0.037 | 0.539 | -0.059 | 0.038 | 0.585 |
| Fornix(Cres)/ Stria Terminalis | -0.014 | 0.033 | 0.847 | -0.013 | 0.031 | 0.788 | -0.022 | 0.034 | 0.816 |
| Internal Capsule | -0.022 | 0.035 | 0.847 | -0.027 | 0.034 | 0.645 | -0.017 | 0.036 | 0.891 |
| Inferior- Fronto-Occipital Fasciculus | -0.001 | 0.037 | 0.988 | -0.003 | 0.036 | 0.940 | -0.015 | 0.038 | 0.908 |
| Posterior Corona Radiata | -0.048 | 0.039 | 0.612 | -0.046 | 0.038 | 0.539 | -0.044 | 0.040 | 0.727 |
| Posterior Limb of Internal Cpasule | -0.018 | 0.034 | 0.847 | 0.006 | 0.033 | 0.935 | -0.002 | 0.036 | 0.948 |
| Posterior Thalamic Radiation | -0.073 | 0.035 | 0.311 | -0.079 | 0.035 | 0.300 | -0.075 | 0.036 | 0.509 |
| Retrolenticular part of Internal Capsule | -0.012 | 0.033 | 0.848 | -0.046 | 0.032 | 0.539 | -0.011 | 0.034 | 0.908 |
| Superior Corona Radiata | -0.017 | 0.040 | 0.847 | -0.020 | 0.039 | 0.753 | -0.004 | 0.042 | 0.948 |
| Superior Fronto-Occipital Fasciculus | -0.073 | 0.038 | 0.311 | -0.023 | 0.036 | 0.743 | -0.067 | 0.039 | 0.513 |
| Superior Longitudinal Fasciculus | -0.079 | 0.039 | 0.311 | -0.064 | 0.038 | 0.469 | -0.056 | 0.041 | 0.585 |
| Sagittal Striatum | -0.065 | 0.035 | 0.319 | -0.062 | 0.035 | 0.469 | -0.074 | 0.037 | 0.509 |
| Uncinate Fasciculus | -0.019 | 0.039 | 0.847 | -0.035 | 0.039 | 0.645 | -0.037 | 0.040 | 0.727 |
| Body Corpus Callosum | -0.001 | 0.041 | 0.988 | -0.035 | 0.041 | 0.645 | -0.015 | 0.042 | 0.908 |
| Corpus Callosum | -0.019 | 0.037 | 0.847 | -0.047 | 0.037 | 0.539 | -0.032 | 0.039 | 0.748 |
| Fornix (column and body) | -0.002 | 0.038 | 0.988 | -0.003 | 0.036 | 0.940 | -0.004 | 0.039 | 0.948 |
| Genu Corpus Callosum | -0.057 | 0.037 | 0.513 | -0.083 | 0.037 | 0.300 | -0.072 | 0.039 | 0.509 |
| Splenium of Corpus Callosum | -0.006 | 0.032 | 0.982 | -0.015 | 0.032 | 0.753 | -0.008 | 0.033 | 0.914 |

*Notes*: All models are adjusted for covariates- age, sex, study site and lab batch effects.

**Supplementary References**

1 Habota T, Sandu A-L, Waiter GD, McNeil CJ, Steele JD, Macfarlane JA *et al.* Cohort profile for the STratifying Resilience and Depression Longitudinally (STRADL) study: A depression-focused investigation of Generation Scotland, using detailed clinical, cognitive, and neuroimaging assessments. *Wellcome Open Res* 2019. doi:10.12688/wellcomeopenres.15538.1.

2 Green C, Shen X, Stevenson AJ, Conole ELS, Harris MA, Barbu MC *et al.* Structural brain correlates of serum and epigenetic markers of inflammation in major depressive disorder. *Brain Behav Immun* 2021; **92**: 39–48.

3 Dale A, Sereno M, Fischl B, Marrett S, Liu A, Halgren E *et al.* FreeSurfer Manual. *Neuroimage* 2002.

4 Desikan RS, Ségonne F, Fischl B, Quinn BT, Dickerson BC, Blacker D *et al.* An automated labeling system for subdividing the human cerebral cortex on MRI scans into gyral based regions of interest. *Neuroimage* 2006. doi:10.1016/j.neuroimage.2006.01.021.

5 Mori S, Van Zijl P, Tamminga CA. Human white matter atlas. *Am J Psychiatry* 2007. doi:10.1176/ajp.2007.164.7.1005.
